# A Regulatory Pathway, Ecdysone-Transcription Factor Relish-Cathepsin L, Is Involved in Insect Fat Body Dissociation

**DOI:** 10.1371/journal.pgen.1003273

**Published:** 2013-02-14

**Authors:** Yao Zhang, Yu-Xuan Lu, Jian Liu, Cui Yang, Qi-Li Feng, Wei-Hua Xu

**Affiliations:** 1 State Key Laboratory of Biocontrol and Institute of Entomology, School of Life Sciences, Sun Yat-Sen University, Guangzhou, China; 2 Department of Life Sciences, School of Biotechnology and Food Engineering, Hefei University of Technology, Hefei, China; 3 Guangdong Provincial Key Laboratory of Biotechnology for Plant Development, School of Life Sciences, South China Normal University, Guangzhou, China; New York University, United States of America

## Abstract

Insect fat body is the organ for intermediary metabolism, comparable to vertebrate liver and adipose tissue. Larval fat body is disintegrated to individual fat body cells and then adult fat body is remodeled at the pupal stage. However, little is known about the dissociation mechanism. We find that the moth *Helicoverpa armigera* cathepsin L (Har-CL) is expressed heavily in the fat body and is released from fat body cells into the extracellular matrix. The inhibitor and RNAi experiments demonstrate that Har-CL functions in the fat body dissociation in *H. armigera*. Further, a nuclear protein is identified to be transcription factor Har-Relish, which was found in insect immune response and specifically binds to the promoter of Har-CL gene to regulate its activity. Har-Relish also responds to the steroid hormone ecdysone. Thus, the dissociation of the larval fat body is involved in the hormone (ecdysone)-transcription factor (Relish)-target gene (cathepsin L) regulatory pathway.

## Author Summary

Insect fat body is the intermediary metabolism organ and the main source of hemolymph components, and it is crucial for insect development and metamorphosis. However, molecular mechanism for the fat body remodeling is almost unknown other than in *Drosophila melanogaster*. A pupal diapause species the cotton bollworm, *Helicoverpa armigera* (Har), is a useful model to study individual or tissue remodeling, because larval fat body will remain integral in diapause-type pupae for months, whereas the dissociation of larval fat body will start on day 0 after pupation in nondiapause-type ones. Here, we find that *H. armigera* cathepsin L (Har-CL) is released from fat body cells into the extracellular matrix for tissue dissociation. A nuclear protein is identified to be transcription factor Har-Relish, which regulates the promoter activity of Har-CL gene. Har-Relish also responds to the steroid hormone ecdysone. Thus, a new regulatory mechanism, ecdysone-Relish-cathepsin L signaling pathway, is involved in the larval fat body dissociation.

## Introduction

In holomatabolous insects, larva undergoes a complete transformation during metamorphosis to form adult. This transformation is accomplished by the destruction of larval tissues and organogenesis of the adult tissues, and is called as tissue remodeling. The extracellular matrix (ECM), which functions in cell adhesion, cell signaling, and the structural maintenance of tissues, must be degraded during tissue remodeling. The ECM alteration is important for embryogenesis, metamorphosis, and cell migration, and it is also degraded during the course of many diseases, for example, cancer growth and metastasis [1], [2]. Two protein families, matrix metalloproteinases and cysteine proteases, are involved in degradation of ECM and intercellular protein from bacteria to mammals [1]–[3], especially cysteine protease cathepsins in cancer.

Previous studies demonstrated that metamorphosis in insects is developmentally regulated by the steroid hormone 20-hydroxyecdysone (20E or ecdysone), the ecdysone binds to its receptors EcR and USP, and mediates a cascade gene expression to promote metamorphosis process, including tissue remodeling [4]. The insect fat body is an important organ, comparable to vertebrate liver and adipose tissue, which performs a myriad of metabolic activities including intermediary metabolism and the homeostatic maintenance of hemolymph proteins, lipids, and carbohydrates [5], [6]. Moreover, fat body also contributes to developmentally specific metabolic activities that produce, store, or release components central to the prevailing nutritional requirements or metamorphic events of the insect [6]. Recently, molecular regulatory mechanism showed that fat body can regulate growth and development through mediating release of the brain hormone [7], [8]. Therefore, understanding the fat body remodeling is crucial for insect development and metamorphosis, and the fat body dissociation is the first step to understand the remodeling of the fat body.

The fat body is made up of a single layer of cells that are encased by a thin basement membrane and forms sheets of tissue. The dissociation of larval fat body involves extensive proteolysis, which makes proteases to degrade basement membrane and ECM between fat body cells, and then causes release of individual fat body cells into hemolymph. An insect cysteine protease, hemocyte cathepsin B has been suggested to participate in the dissociation of larval fat body in Dipteran species, *Sarcophaga peregrine* [9], [10]. This 29 kD cathepsin was excreted from pupal hemocytes, bound to the basement membrane of larval fat body, and led to the fat body dissociation. Rabossi *et al.* observed the hemocyte binding to the fat body of another Dipteran, *Ceratitis capitata*, and an aspartyl protease was purified from *C. capitata* fat body [11]. The temporal activity profile of the enzyme during metamorphosis was correlated well with the fat body dissociation, but it is unclear whether the aspartyl protease was derived from the fat body or hemocyte. Hori *et al.* [12] and Kobayashi *et al.* [13] suggested that a 200 kD hemocyte-specific recognition protein could interact with the fat body to trigger the release of cathepsin B through an unknown mechanism. However, the 200 kD recognition protein was demonstrated to be myosin heavy chain derived from degraded larval muscle, but not from pupal hemocyte [14]. These results implied that the fat body dissociation is done by a certain internal factor.

In *Drosophila melanogaster*, the matrix metalloproteinases 2 (MMP 2) was related to histolysis of larval tissues, proventriculus and gastric caeca, but not fat body [15]. Recently, Nelliot *et al.* elegantly demonstrated that fat body remodeling in *D. melanogaster* is a hemocyte independent process based on a strategy to ablate the hemocytes by ectopically expressing a cell death gene *bead involution defective* [16]. Bond *et al.* [17] proved that βFTZ-F1 is involved in *Drosophila* fat body remodeling by the regulation of the MMP2 expression. Obviously, fat body dissociation is caused by an internal factor, but not hemocyte. However, little is known about the mechanism for the fat body dissociation other than *Drosophila*.

In previous study, we showed an important role for cysteine protease cathepsin L in larval moulting of the cotton bollworm *Helicoverpa armigera* [18]. In whole-body larvae and larval hemolymph, the activity and expression of *H. armigera* cathepsin L (Har-CL) was low after the larval ecdysis (4^th^–5^th^ instar and 5^th^–6^th^ instar) and increased significantly before next moulting, which suggests that Har-CL is regulated strictly in larval development through degradation of ECM for larval moulting. However, a major difference of expression and activity of Har-CL between whole body and hemolymph was found in day 0 pupae. In hemolymph, Har-CL expression and activity in day 0 pupae was much lower than in day 5 of sixth instar larvae. In contrast, Har-CL expression in day 0 whole body pupa was comparable to that of day 5 of sixth instar larvae. The difference may be the result of high Har-CL expression in a certain tissue other than the hemolymph, such as fat body, during early pupal development. If so, Har-CL may be crucial in the dissociation of the larval fat body.

Developmental arrest, called as diapause in insects, is a good model to study individual or tissue development [19]. As a pupal diapause species the moth, *H. armigera*, larval fat body will remain integral in diapause-type pupae for months, whereas the dissociation of larval fat body will start on day 0 after pupation in nondiapause-type ones. *H. armigera*, therefore, is a suited animal to study the fat body dissociation. In the present paper, we study the activity and expression of Har-CL in nondiapause- and diapause-destined *H. armigera* individuals. These results show that Har-CL in day 0 of nondiapause-type pupae is released into the extracellular matrix of the fat body for its tissue dissociation, but not in diapause-type pupae. The inhibitor and RNAi experiments demonstrate clearly that Har-CL functions in the fat body dissociation. Further, transcription factor Har-Relish, which is a member of NF-κB family and functions in the immune response through regulating antimicrobial peptide gene expression in insects, specifically binds to the promoter of Har-CL to regulate its activity. Har-Relish transcription can respond to ecdysone *in vivo*. Thus, a new regulatory mechanism, ecdysone-Relish-cathepsin L signaling pathway, is involved in the larval fat body dissociation.

## Results

### Changes of proteolytic activity in fat body

**Figure 1:**
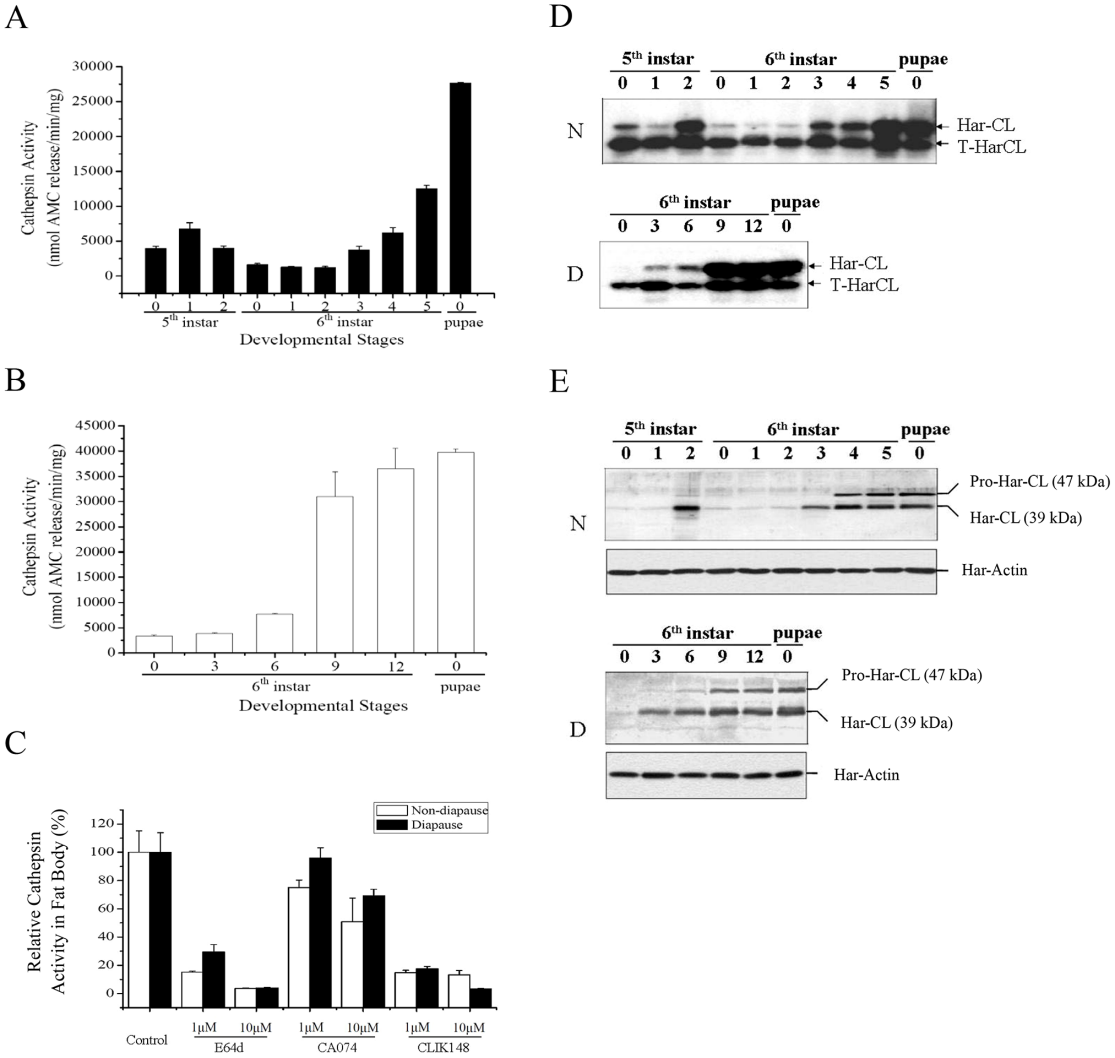
Developmental changes of *Helicoverpa armigera* cathepsin L (Har-CL) proteolytic activity, mRNA, and protein in fat body. Proteolytic activity in the fat bodies of nondiapause- (A) and diapause-destined individuals (B). Protein extracted from the fat body of the fifth or sixth instar larvae to new pupae was used to measure total proteolytic activity against Z-Phe-Arg-MCA. Proteolytic activity was presented as nmol of fluorescent aminomethylcourmarin (AMC) released per minute per mg protein of the fat body. (C) Inhibition of proteolytic activity by E-64, CA-074 and CLIK148 in the fat body of day 0 pupa. Inhibitors and their concentrations are indicated in the legend. The results were presented as per cent (%) of proteolytic activity, defined in (A), relative to untreated controls. Each column represents the mean ± SD of three separate experiments in A, B, and C. Developmental changes of Har-CL mRNA (D) and protein (E) from the fifth or sixth instar larvae to new pupae in nondiapause type (N) and diapause type (D). Har-CL mRNA was detected by semi-PCR that truncated Har-CL (T-HarCL) mRNA (1 ng) and total RNA (1 µg) was reverse-transcribed, PCR-amplified with 24 cycles, and subjected to Southern blot analysis. The truncated-Har-CL is as internal control for semi-quantitative PCR. The numbers in the X axis represent days of the 5th and 6th instar larvae. Har-CL protein was detected by Western blot that fat body protein (20 µg) from each stage were separated, and transferred to membrane and hybridized by using Har-CL polyclonal antibodies.

Using a synthetic substrate of cathepsins B and L, Z-Phe-Arg-methyl- coumarylamide (Z-F-R-MCA), we examined the pattern of proteolytic activity in the fat body from the fifth instar larvae to new pupae. In the fifth instar larvae of nondiapause-destined, activity was low on day 0, and then increased on day 1, followed by a gradual decline (Figure 1A). During the early development (day 0–2) of the sixth instar larvae, proteolytic activity decreased continually, reaching the lowest point on day 2. Activity then increased progressively until pupation and reached a peak in day 0 pupae. In diapause-destined individuals, the trend of activity was similar to nondiapause type (Figure 1B). However, activities in the fat body of diapause-type individuals were much higher than that of nondiapause-type ones.

To determine the relative contribution of cathepsin B or L activity in fat body of day 0 pupae, we added the cysteine protease broad-range inhibitor E-64, the cathepsin B-selective inhibitor CA074 or the cathepsin L-selective inhibitor CLIK148 to the Z-F-R-MCA reaction mixture. In nondiapause- or diapause-destined pupae, 10 µM E-64 or CLIK148 inhibited more than 90% of the Z-F-R-MCA proteolytic activities from the fat body, while cathepsin B-selective inhibitor CA074 was much less potent than E-64 or CLIK148 (Figure 1C). These observations suggest that Har-CL is the major cysteine protease in the fat body.

### Har-CL expression in fat body

Using a combination of competitive RT-PCR and Southern blot analysis and Western blot, the temporal patterns of Har-CL mRNA and protein from the fifth instar larvae to new pupae were analyzed in nondiapause-destined individuals. Har-CL mRNA was barely detected on day 0 and 1 of the fifth instar, but the mRNA levels increased sharply to a peak on day 2 (Figure 1D). The expression then declined to low levels during days 0–2 of the sixth instar larvae, and increased gradually again to the second peak at the end of the sixth larval instar and day 0 pupae. The Western blot showed a similar pattern to changes of Har-CL mRNA and a 47 kDa propeptide and a 39 kDa mature peptide were detected in the fat body, especially high protein expression at late stage of the sixth instar larvae and new pupae (Figure 1E). In the diapause-destined individuals, similar patterns of Har-CL mRNA (Figure 1D) and protein (Figure 1E) expression were observed. These results showed clearly a high expression of Har-CL in day 0 pupal fat body, implying that Har-CL may exert its effect on the fat body dissociation.

### Localization of Har-CL in the fat body

**Figure 2:**
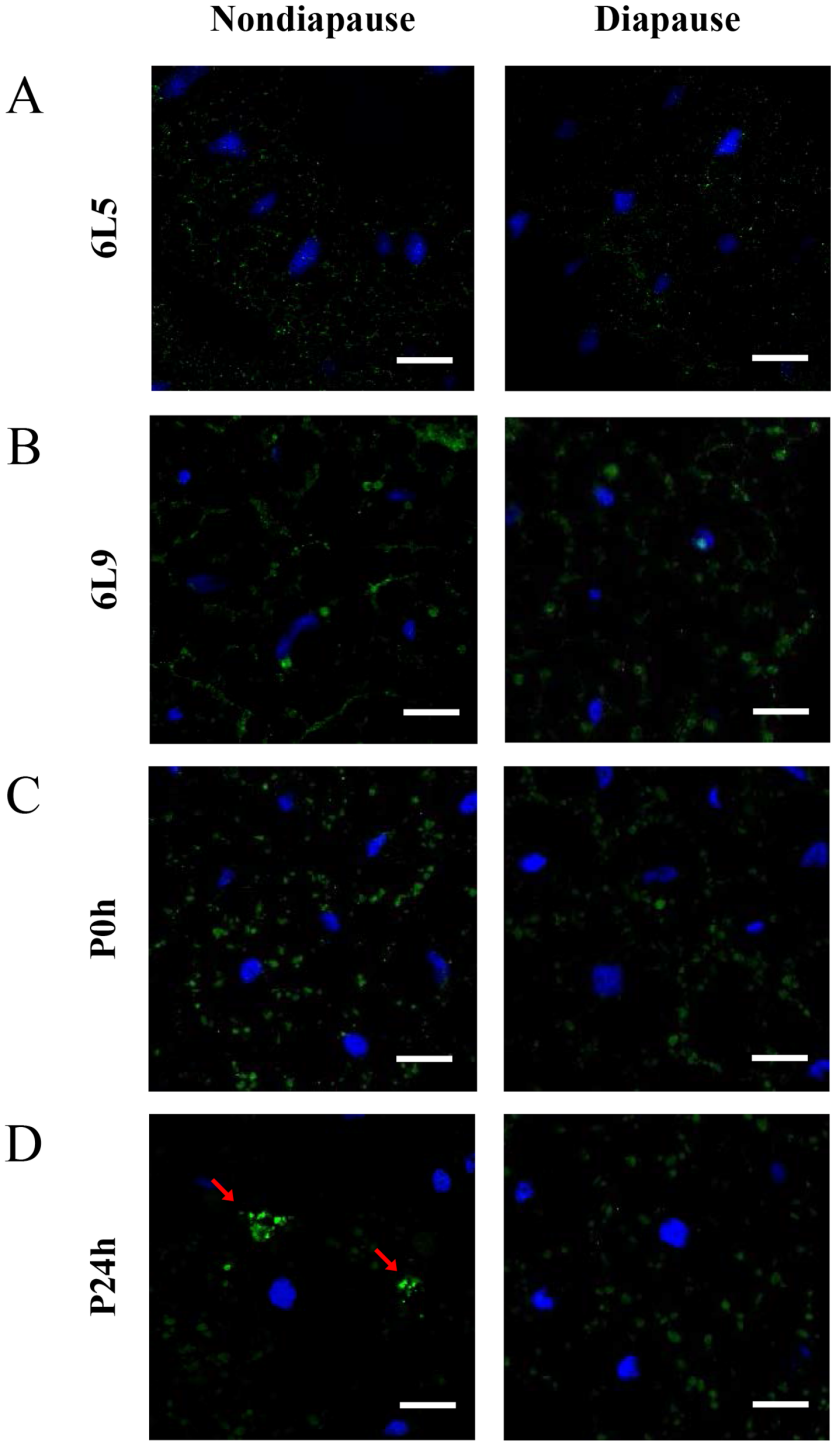
Immunohistochemical analysis of Har-CL in the fat body. This figure is from Figure S1, and the panes in Figure S1 are magnified. Har-CL positive signals (green) are localized in the fat body of nondiapause- and diapause-destined individuals, and the nucleus were stained with blue. Fat bodies from day 5 (6L5, feeding stage) (A) and day 9 (6L9, pre-pupal stage) (B) of the 6^th^ instar larvae; Fat bodies from 0 h (P0h) (C) and 24 h (P24h) (D) after pupation. Bar = 25 µm.

Using immunocytochemical methods, we investigated the distribution of Har-CL in the fat bodies of diapause- and nondiapause-destined individuals. To compare accurately the difference of Har-CL expression between diapause- and nondiapause-destined individuals, both two type larvae were reared in the same temperature (20°C) with long day (nondiapause type) or short day (diapause type) to synchronize developmental time. In feeding 6^th^ instar larvae (day 5) and pre-pupae (day 9), Har-CL-positive signals were similar in the fat bodies of diapause- and nondiapause-destined individuals (Figure 2A and 2B), but a significant increase was found on day 9 in both type of fat body cells. These observations are consistent with high levels of Har-CL expression and activity at the late stage of the sixth instar larvae (Figure 1). However, a significant difference of Har-CL expression in the fat body was seen in day 0 pupae. The amounts of Har-CL-positive signals in the fat body of nondiapause-destined pupae were similar with that of diapause-destined ones in 0 h after pupation, but the intensities of Har-CL signals were more clear in nondiapause-destined ones (Figure 2C). The result implied that Har-CL was moved to the plasma membrane of the fat body cells in nondiapause-destined individuals, and Har-CL in diapause-destined ones still remained in the middle of fat body cells. In 24 h after pupation, Har-CL from lysosomes of the fat body cells were released in nondiapause-type individuals (Figure 2D), whereas those Har-CL still remained in lysosomes of the fat body and randomly distributed in cytoplasm in diapause-type ones as the same with 0 h after pupation. We also did the fat body of diapausing individuals (day 20 after pupation, Figure S1), which is similar with day 0 pupae.

To demonstrate whether Har-CL from lysosomes of the fat body cells releases into the hemolymph in nondiapause type pupae on day 0, we detected Har-CL protein and activity in the hemolymph and fat body. The result showed that both Har-CL protein and activity in hemolymph of day 0 pupae are very less or low compared with the fat body (Figure S2), suggesting that Har-CL protein from the fat body is not released into hemolymph. Therefore, Har-CL protein from the fat body of nondiapause-destined pupae is likely released into the ECM of fat body cells, and is closely correlated with the fat body dissociation.

### Har-CL function on the fat body dissociation

**Figure 3:**
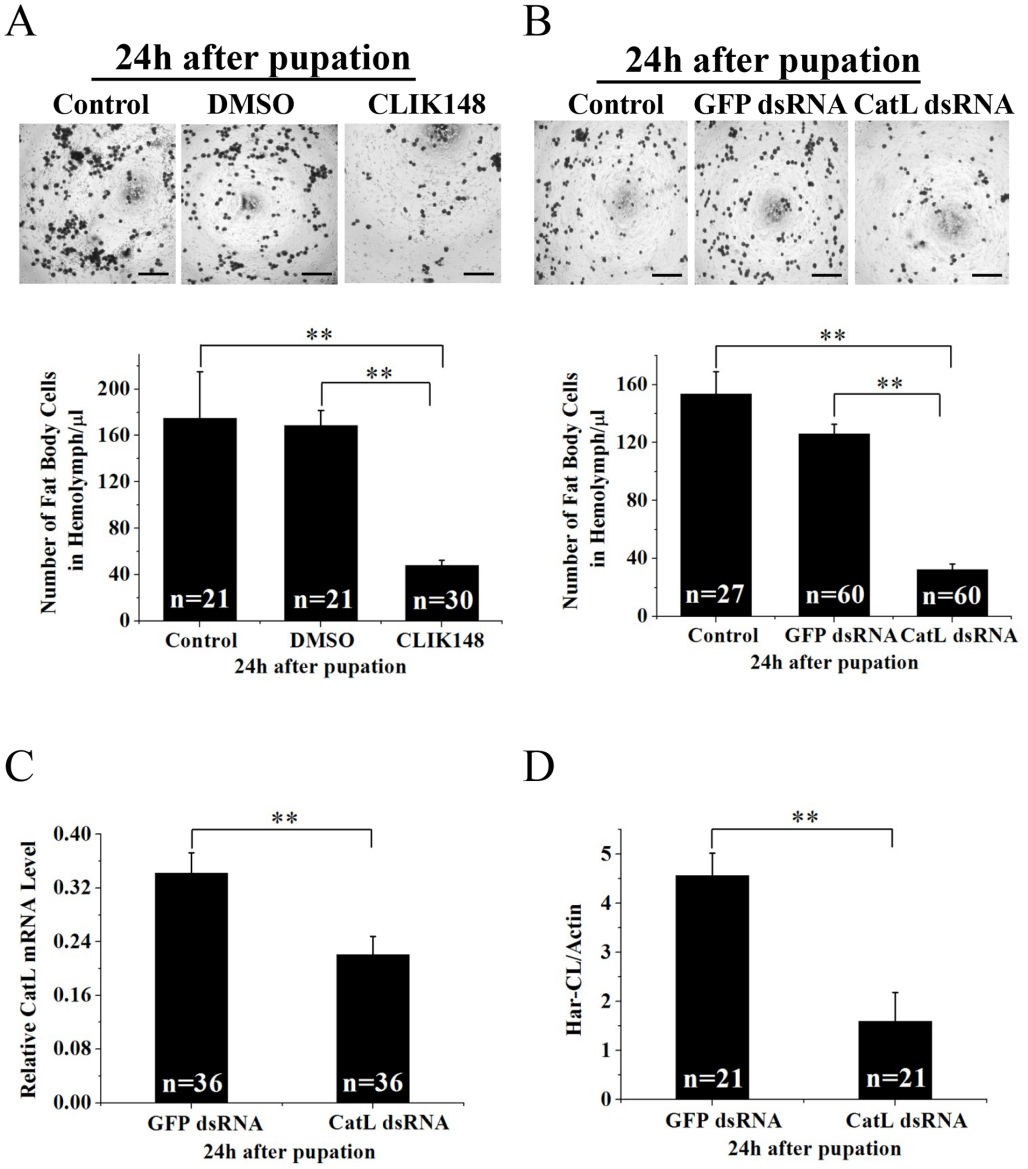
Effects of inhibitor and dsRNA on the fat body dissociation. (A) New pupae 2 h after pupation were injected with CLIK148, and counted the dissociated fat body cells in hemolymph 24 h after pupation as described in Figure S2. Scale bar is 200 µm. (B) *H. armigera* larvae were injected with dsRNA against Har-CL (15 µg) 18 h before pupation. The dissociated fat body cells in hemolymph were detected 24 h after pupation, equaling to 42 h after injection. The fat body cells in hemolymph were counted and injection of *GFP* dsRNA (15 µg) was as the control. Scale bar is 200 µm. Error bars represent S.D.s that were obtained from three independent experiments, the “n” represents number of individuals. RNAi knockdown efficiency in the fat body injected with Har-CL dsRNA were detected with qRT-PCR (C) and Western blot (D). Har-CL and GFP dsRNAs was respectively injected into last day of the sixth instar around 18 h before pupation, RNA and protein were respectively extracted 24 h after pupation. Error bars represent S.D.s that were obtained from three independent experiments.

We first investigated the numbers of the fat body cells in hemolymph from 6 h to 24 h after pupation, the dissociated numbers of the fat body cells were less in 6 h and 12 h, increased significantly in 18 h, and reached heavily dissociation in 24 h (Figure S3). We then injected specific inhibitor CLIK 148 (solution in 1% DMSO) for Har-CL into new pupae 2 h after pupation. Compared with a control injected with 1% DMSO, the dissociated fat body cells in hemolymph were significantly less with inhibitor in 24 h after pupation (Figure 3A), indicating that Har-CL plays a key role in the dissociation of the larval fat body. Further, we injected dsRNA directly against Har-CL or GFP into nondiapause-type larvae on the last day of the sixth instar, approximately 18 h before pupation, and the dissociated fat body cells in hemolymph and Har-CL expression in the fat body at the mRNA and protein levels were investigated in 24 h after pupae. The programmed fat body dissociation was significantly suppressed by injection of dsRNA against Har-CL, compared with a control that injected with the GFP dsRNA (Figure 3B). Har-CL mRNA (Figure 3C) and protein (Figure 3D) in the fat body were detected by real-time PCR and Western blot. Both Har-CL mRNA and protein had a significant decrease by RNAi, compared with the control injected with the GFP dsRNA. This result showed that dsRNA causes a decrease of the dissociated fat body cells in hemolymph through knock-down of Har-CL expression. Taken together, Har-CL indeed functions in the dissociation of the larval fat body.

### Characterization of 5′-upsteam regulative region of Har-CL gene

To characterize the regulatory mechanism of Har-CL gene expression at the transcription level, a 1920-bp fragment from the 5′-upsteam region of Har-CL gene was cloned using the genome walking technique that was described in the Materials and Methods section, and the cloned region of Har-CL gene was subsequently sequenced. The potential consensus sequences for regulatory elements in the promoter were analyzed using the TFSEARCH website (http://www.cbrc.jp/research/db/TFSEARCH.html) [20], and several possible transcription factor-binding sites were shown in Figure S4. These sites include POU, CdxA, MyoD, BR-C Z, NF-κB, E-box, and GATA-1.

**Figure 4:**
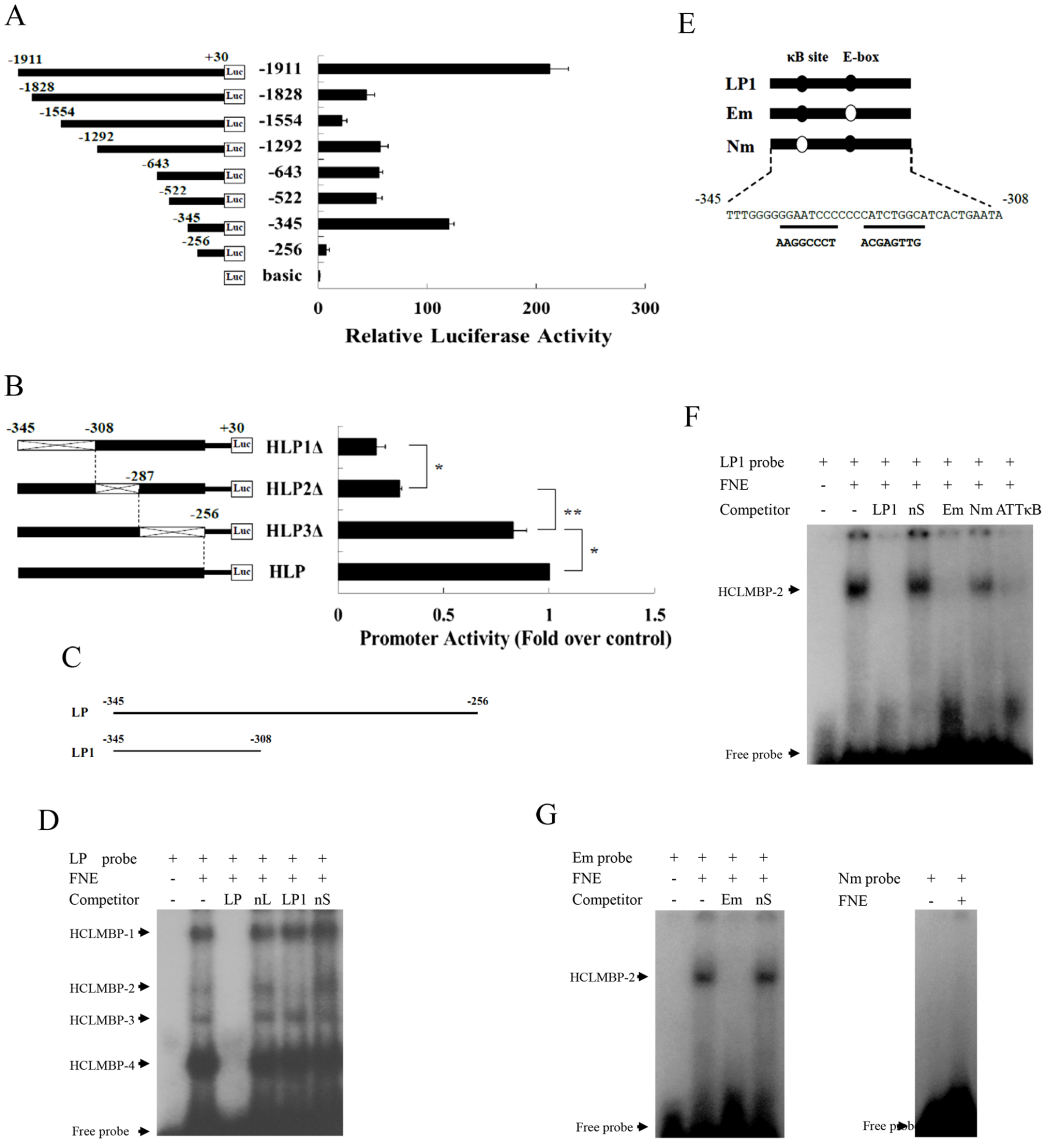
Identification of a transcription factor, HCLMBP-2, which may be a member of the NF-κB protein family. (A) A series of deletion constructs that contained different lengths of possible Har-CL promoters were fused to luciferase reporter gene, and the constructed plasmids were transfected into HzAM1 cells. The pGL3-basic plasmid was used as a negative control because it has no promoter sequence. (B) Three deletion mutations (HLP1-3Δ) in the active region (−345∼−256) of the promoter were cloned into reporter plasmid, and the constructed plasmids were used in transfection experiments that were aimed at detecting promoter activity. The cross indicates the deletion mutations in the regions (−345∼−308, −308∼−287 and −287∼−256). Error bars represent S.D.s that were obtained independently from three separate experiments; * indicates a *p* value of <0.05; ** indicates a *p* value of <0.01; *p* values were determined using a paired one-sample *t*-test. (C) Schematic drawing of the relative positions of probes LP and LP1 spanning the activating region. (D) Five µg of nuclear protein extracts from the fat bodies of the sixth instar larvae were used in each reaction. Probe LP was incubated with nuclear protein extracts in the absence of a competitor (lane 2) or in the presence of a 100-fold concentration of a possible competitor, including unlabeled LP, nL, LP1, and nS (lanes 3–6). FNE, fat body nuclear extracts; nL, non-specific long competitor; nS, non-specific short competitor. The shift bands are indicated with arrows. HCLMBP-1–4, Har-CL modulating binding protein 1–4. (E) Schematic representation of two probes that were mutated at either the NF-κB-binding site or the E-box site. Probes Em and Nm were designed for E-box and NF-κB-binding site mutations, respectively. Mutant regions were underlined and the resulting sequences following mutation are listed below the underlined sequences. (F) Five µg of nuclear protein extracts from the fat bodies of the sixth instar larvae were used in each reaction. The radiolabeled probe LP1 was used in the initial EMSA, and 100-fold excesses of unlabeled LP1, nS, Em, Nm, and ATTκB probes were used in competition EMSAs (lanes 3–7). ATTκB, *B. mori* NF-κB-binding sequence. (G) Em (left) and Nm (right) were used as probes in EMSAs. A 100-fold excess of either a specific probe (Em) or a non-specific probe (nS) was used for competition analysis. HCLMBP-2 was indicated with an arrow.

We first cloned eight truncated promoter sequences of Har-CL gene into a pGL3-basic luciferase reporter vector, after which we measured promoter activity to confirm that the potential *cis*-elements were involved in the regulation of Har-CL transcription (Figure 4A). These constructs were co-transfected into HzAM1 cells, which originate from the ovaries of *Helicoverpa zea* [21], using the pRL-TK plasmid as an internal control to determine the transfection efficiency. Promoter activity was measured using a dual-luciferase reporter system, and two strong luciferase activity signals were detected when the HzAM1 cells were transfected with the +30 to −345 bp and +30 to −1911 bp segments, respectively.

To identify *cis*-elements for the promoter activity that were located between −345 and −256 bp, several deletion constructs were generated as follows: HLP1Δ between −345 and −308, HLP2Δ −308 and −287, and HLP3Δ between −287 and −256 (Figure 4B). The promoter HLP (+30∼−345) had the highest activity level, whereas the promoter activity that was associated with the HLP1Δ and HLP2Δ constructs was dramatically reduced. This finding suggests that −345∼−308 and/or −308∼−287 regions are important for activating the transcription of Har-CL gene.

Electrophoresis mobility shift assay (EMSA) was performed with the nuclear extracts from *H. armigera* fat bodies to further characterize the potential transcription factor activity that mediated the activator domain of Har-CL gene, and the probe LP was designed according to the −345∼−256 region, which spans the activation region of Har-CL promoter (Figure 4C). The labeled probe (LP) bound to four different complexes, which were termed Har-CL modulating binding proteins 1–4 (HCLMBP-1–4) (Figure 4D). These DNA-protein interactions were specific; the specific probe sL (unlabeled LP) competes for binding sites with the HCLMBPs, whereas a non-specific probe nL does not. HCLMBP-2 was also able to bind the probe LP1 (−345∼−308), which can be inferred based on the observation that the HCLMBP-2 band could not be detected when the unlabeled probe LP1 was used as a competitor. As a control, a non-specific probe nS of the same length could not compete with probe LP1. Thus, LP1 interacts with a specific transcription factor.

### HCLMBP-2 may be a member of the NF-κB family

After investigating the LP1 sequence, we deduced the presence and locations of two *cis*-elements, an E-box and an NF-κB-binding site, in the segment. We designed two mutations, one of which was located in the E-box (Em) and one of which was located at the NF-κB-binding site (Nm). These are shown in Figure 4E. HCLMBP-2 could be detected using probe LP1, and unlabeled probe Em could effectively bind to HCLMBP-2 in a competitive manner. However, the probe Nm failed to bind competitively to HCLMBP-2 in the presence of probe LP1 (Figure 4F). This result indicates that the HCLMBP-2 may bind to the NF-κB-binding site in LP1. To further confirm the location of the HCLMBP-2-binding site, we prepared a probe from a *Bombyx mori* NF-κB-binding sequence that was called ATTκB [22], [23]. That probe could effectively and competitively inhibit the binding of HCLMBP-2 to LP1.

Furthermore, a competition EMSA was performed using probes Em and Nm. The presence of HCLMBP-2 was still detected when the probe Em was used, and unlabeled Em could bind competitively with HCLMBP-2. However, no evidence that HCLMBP-2 bound to the Nm was found (Figure 4G). Thus, it appears that HCLMBP-2 binds to the NF-κB site but not to the E-box, which suggests that HCLMBP-2 may belong to the NF-κB transcription factor family.

### Cloning and characterization of the NF-κB transcription factors *Relish* and *Dorsal*

To obtain the cDNA sequence of the NF-κB transcription factors, we designed several pairs of degenerate primers for *Relish* and *Dorsal* based on the *Relish* and *Dorsal* sequences in *D. melanogaster* and *B. mori*. A 356-bp fragment for *Relish* and a 332-bp fragment for *Dorsal* were amplified via RT-PCR. The amino acid sequences that were deduced from this procedure showed high degrees of homology with known *Relish* and *Dorsal* sequences, respectively. Therefore, the specific primers RR1R, RR2R, RR1F, and RR2F (for *Relish*) and the primers DR1R, DR2R, DR1F, and DR2F (for *Dorsal*) were subjected to 5′- and 3′-rapid amplification of the cDNA end (RACE) according to the two cDNA sequences that had been obtained from the partial amplification. The two entire cDNAs that encoded *Relish* (GenBank No. JN315690) and *Dorsal* (GenBank No. JN315687) in *H. armigera* were obtained using this procedure.

**Figure 5:**
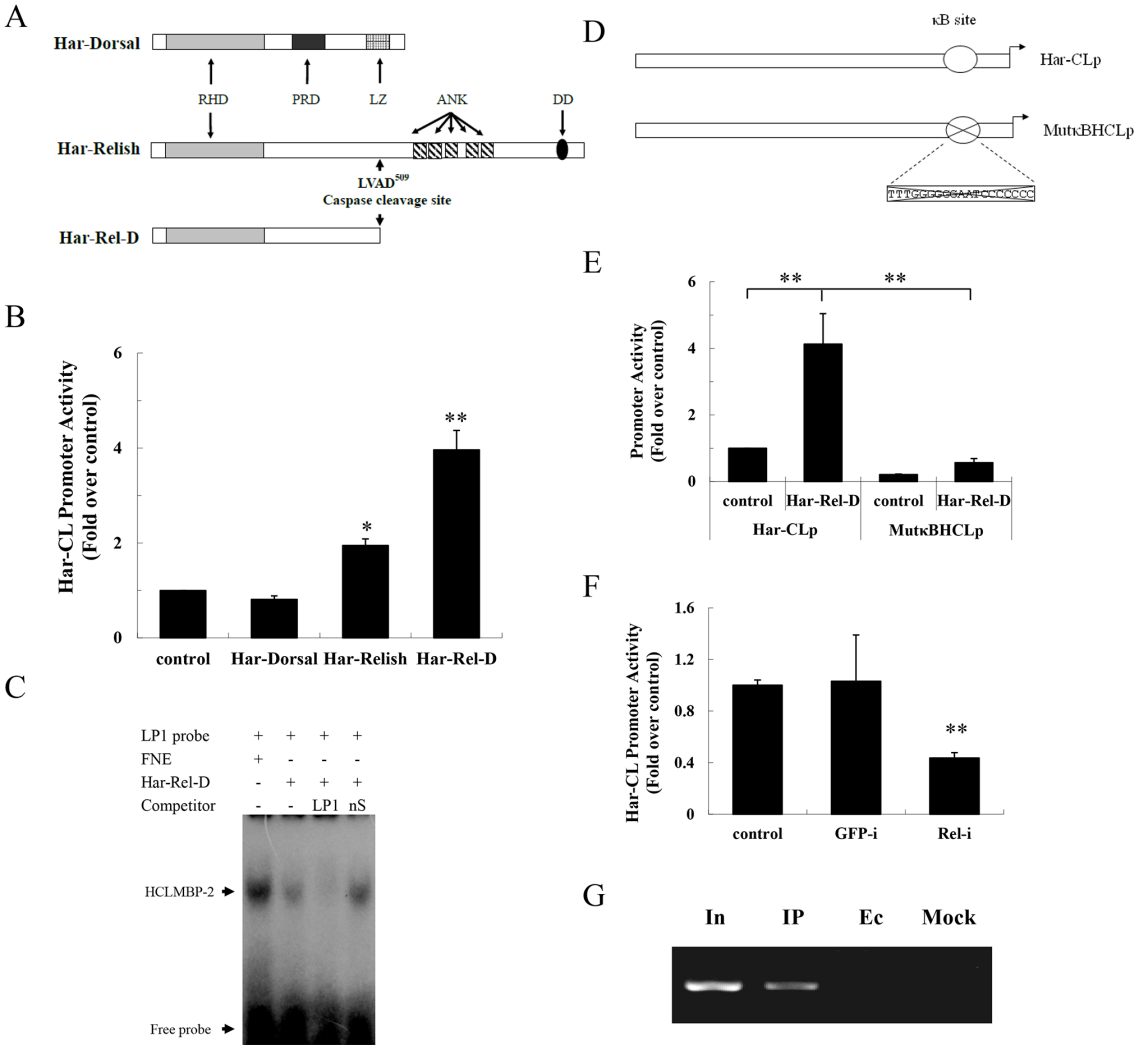
Har-Relish binding to the NF-κB-binding site and increasing Har-CL promoter activity. (A) Schematic representations of Har-Dorsal, Har-Relish, and Har-Rel-D. Har-Rel-D is a mature molecule that is derived from the cleavage of Har-Relish at the 509 aa residue. RHD, Relish homology domain; ANK, ankyrin repeat domain; PRD, proline-rich domain; LZ, leucine zipper motif; DD, death domain. (B) Luciferase activity assay for Har-CL promoter. Each reporter plasmid (200 ng) was transfected into HzAM1 cells in conjunction with either pIZ/V5-His plasmids, which were used as a negative control, or with recombinant Har-Dorsal, Har-Relish or Har-Rel-D plasmids. The control represents the transfection with only Har-CL promoter and the pIZ/V5-His plasmid. (C) Detection of Har-Rel-D binding activity by EMSA. FNE, fat body nuclear extracts as a positive control (lane 1); Har-Rel-D, *in vitro-*translated protein (lane 2); LP1, specific probe (lane 3); nS, non-specific competitor (lane 4). (D) Schematic representation of the mutated NF-κB-binding site in Har-CL promoter (MutκBHCLp). The cross indicates the deletion of the NF-κB-binding site. (E) Luciferase activity assays for Har-CL promoter (Har-CLp) and the mutated Har-CL promoter (MutκBHCLp). Recombinant Har-Rel-D plasmid that contained either Har-CLp or MutκBHCLp were transfected into HzAM1 cells. The pIZ/V5-His plasmid was used in conjunction with either Har-CLp or MutκBHCLp as a control. (F) Luciferase activity assay of Har-CLp. Har-Relish expression was knocked down by Relish dsRNA (Rel-i), and the GFP dsRNA (GFP-i) was used as a control. The control represents no addition of dsRNA. Error bars represent S.D.s that were obtained from three independent experiments; * indicates a *p* value of <0.05, and ** indicates a *p* value <0.01; *p* values were determined using paired one-sample *t*-tests. (G) ChIP analysis. In, Input; IP, the DNA immunoprecipitated by anti-Har-Relish; Ec, empty control (immuneprecipitated by pre-immune serum); Mock, mock control (immunoprecipitated by anti-Har-CL).

Har-Relish cDNA contains an open reading frame (ORF) that encodes a protein that is composed of 945 amino acids and that has identity levels of 52%, 44%, and 33% with the reported ORFs in the *Relish* genes from *B. mori* (GenBank No. NP 001095935), *D. melanogaster* (GenBank No. AAF20133) and *A. aegypti* (GenBank No. AAM97895), respectively. The RHD and ANK domains of Har-Relish are conserved with these domains in the other three species (Figure S5A-a and S5B-a). A caspase cleavage site that is located between Asp and Ser at amino acid residues 509∼510 is found in Har-Relish protein (Figure 5A). This site can be cut by Dredd, which is a caspase-8 homolog. In *D. melanogaster*, Dredd cleavage of the Relish resulted in an active form of Relish, Rel-D, and the resulting Rel-D protein enters nucleus to regulate gene transcription [24]. Thus, mature Har-Rel-D might also have a similar post-translational regulatory role.

Har-Dorsal cDNA encodes a protein that is composed of 564 amino acids and that has 51%, 53% and 56% identities with the *Dorsal* ORFs of *B. mori* (GenBank No. NM 001172825), *D. melanogaster* (GenBank No. NM 165217) and *A. aegypti* (GenBank No. XM 001652790), respectively. Har-Dorsal has an RHD domain, a proline-rich region and a leucine zipper, all of which suggest that it also belongs to the NF-κB family (Figure 5A; Figure S5A-b and S5B-b).

### Expression patterns of Har-Relish and Har-CL gene during larval-pupal development

We first performed Northern blots to estimate the size and the alternative splicing of the endogenous *Relish* transcript. The total RNA was extracted from six tissues in the sixth instar larvae, and Har-Relish was detected solely in the fat body, not in the remaining five tissues. The size of the hybridized transcript was approximately 3.4 kb, which was consistent with the predicted size (Figure S6A), and thus, the cloned cDNA most likely represents the full-length Har-Relish mRNA.

The RNA and protein extracts that were obtained from the fat bodies of the sixth instar larvae and new pupae were used to perform a temporal expression analysis. According to the results of competitive RT-PCR procedures, Har-Relish mRNA expression level was lowest on day 1, and it gradually increased until the animal reached the new pupa stage (Figure S6B). This expression pattern is similar to the pattern of *Relish* expression in *D. melanogaster* [25]. A 58-kDa protein band was found using an antibody that was specific for Har-Relish, and this band was identical to that predicted for Har-Rel-D (Figure S6C). Har-Rel-D, which is the active form of Har-Relish, was detected on day 5 of the sixth instar, and gradually increased as the larva progressed towards the new pupa stage. Thus, we have shown that the level of protein expression may correspond to the level of mRNA expression during larval-pupal development.

### Overexpression of Har-Relish can activate Har-CL promoter

To confirm the role(s) of Har-Relish and/or Har-Dorsal in the regulation of Har-CL gene expression, three recombinant plasmids (Har-Relish, Har-Rel-D, and Har-Dorsal) were constructed. We co-transfected HzAM1 cells with Har-CL promoter and the three recombinant plasmids. As shown in Figure 5B, the forced expressions of Har-Relish and Har-Rel-D significantly activated Har-CL promoter, and Har-Rel-D was particularly potent. In contrast, the forced expression of Har-Dorsal from its recombinant plasmid did not result in the activation of Har-CL promoter.

Har-Relish had a regulatory effect on Har-CL gene promoter, and thus, we further examined its binding characteristics to determine whether they matched those of HCLMBP-2. We performed an EMSA in which nuclear extracts were incubated with either the labeled probe LP1 or with *in vitro-*translated Har-Rel-D. Har-Rel-D that had been translated *in vitro* bound to the promoter efficiently, and the binding activity of Har-Rel-D could be competitively reduced by incubation with the unlabeled specific competitor LP1. In contrast, the unlabeled nonspecific competitor nS was not able to compete with the binding of LP1. Figure 5C shows that HCLMBP-2 and Har-Relish have similar DNA sequence-binding specificities. Finally, we constructed a mutation in the NF-κB-binding site of Har-CL promoter (MutκBHCLp); the mutated promoter was then introduced into the reporter plasmid and co-transfected with Har-Rel-D (Figure 5D). The activity of the mutant Har-CL promoter MutκBHCLp, which could not bind Har-Rel-D, was significantly lower than that of the wild-type Har-CL promoter (Figure 5E). When Har-Relish expression in HzAM1 cells was knocked down using dsRNA, the luciferase activity of Har-CL promoter was lower than that in control cells that had been treated with GFP dsRNA (Figure 5F). We used a ChIP assay to measure Har-Relish binding activity to Har-CL promoter so that we could verify whether Har-Relish is able to bind to Har-CL promoter *in vivo*. A positive band that corresponded to Har-CL promoter was detected by PCR when we used the anti-Har-Relish antibody; negative controls were not detected (Figure 5G). All these results support the notion that Har-Relish is the most likely identity of the HCLMBP-2 protein and that this protein binds to Har-CL promoter.

### Relish and cathepsin L can be regulated by ecdysone

**Figure 6:**
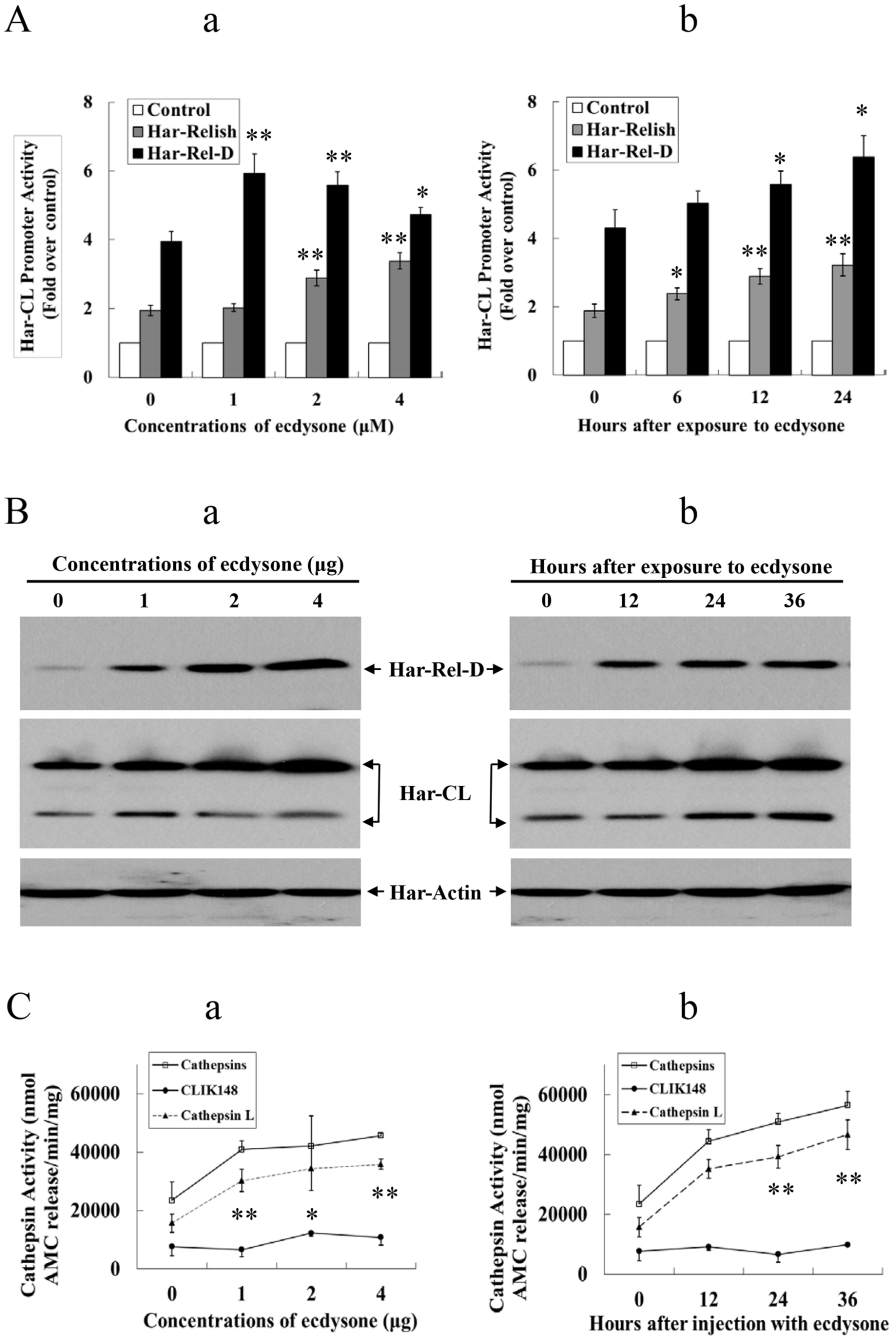
Har-Relish and Har-CL can respond to ecdysone. (A) Har-CL promoter activity in response to ecdysone exposure. (a) Dose-related response to ecdysone administration. To conduct luciferase activity assay, recombinant Har-Relish or Har-Rel-D plasmid was transfected into HzAM1 cells that contained Har-CL promoter (Har-CLp), and the cells were cultured in the presence of 0, 1, 2, or 4 µM ecdysone solution for 12 h. (b) Time-related response to ecdysone administration. HzAM1 cells that contained Har-CLp were transfected with Har-Relish or Har-Rel-D plasmids, after which they were cultured in a medium that contained a 1 µM ecdysone solution for 0, 6, 12 or 24 h. The activity of Har-CLp without ecdysone was used as the control. (B) Har-Relish and Har-CL proteins in response to ecdysone administration. (a) Dose-related response to ecdysone. Various doses of ecdysone (0, 1, 2, and 4 µg) were injected into day 6 of the sixth instar larvae, and protein was extracted from the fat body 12 after injection. (b) Time-related response to ecdysone exposure. Two µg of ecdysone were injected into day 6 of the sixth instar larvae; each injection was followed by a 12 h-interval after which protein was extracted from the fat body. Har-actin was used as a control. (C) Har-CL proteolytic activity in response to ecdysone. (a) Dose-related response to ecdysone. Various quantities of ecdysone (0, 1, 2, and 4 µg) were injected into day 6 larvae of the sixth instar, and protein was then extracted from larval fat body 12 h after injection. (b) Time-related response to ecdysone administration. Two µg of ecdysone was injected into day 6 larvae of the sixth instar, and protein was subsequently extracted from the larval fat body after a 12-h interval. Error bars represent S.D.s that were obtained based on three independent experiments; * indicates a *p* value of <0.05, and ** indicates a *p* value <0.01; *p* values were determined using paired one-sample *t*-tests.

It is well known that ecdysone is one of the most important hormones for metamorphosis and development in insects. Previous studies have demonstrated that ecdysone regulates the expression of Har-CL gene [18], but the mechanism by which it accomplishes this is still unknown. The results that have been described above indicate that Har-Relish can also regulate Har-CL gene expression, and thus, we speculate that ecdysone regulates Har-CL gene expression by activating the transcription factor Har-Relish. To confirm this hypothesis, ecdysone was added into the cell culture medium of HzAM1 cells that had been co-transfected with Har-CL promoter and *Har-Relish* or *Har-Rel-D* recombinant plasmids. The luciferase activities of cells that had been transfected with either *Har-Relish* or *Har-Rel-D* were significantly higher than those of the controls, especially after a 1 µM of ecdysone was added to the culture medium (Figure 6A-a). The luciferase activities of cells that had been co-transfected with both Har-CL promoter and *Har-Relish* or *Har-Rel-D* gradually increased from 6 h to 24 h after cells were exposed to ecdysone (Figure 6A-b). Different doses of ecdysone were injected into the sixth instar larvae on day 6, and the amounts of Har-Relish protein in the fat bodies increased gradually from 1 to 4 µg in a dose-dependent manner (Figure 6B-a). We then injected 2 µg of ecdysone into the sixth instar larvae on day 6, and we found that the concentration of Har-Relish protein gradually increased from 12 h to 36 h after injection (Figure 6B-b). The results of these *in vivo* experiments clearly show that Har-Relish responds to exposure to ecdysone.

Finally, we also investigated the expression and activity levels of Har-CL protein when ecdysone was injected into the sixth instar larvae on day 6. As the same as Har-Relish above, Har-CL proteins in the sixth instar larvae had increased significantly after injection of 1- to 4-µg of ecdysone, and when 2 µg of ecdysone were injected into the larvae, the amount of Har-CL protein in the fat body gradually increased from 24 to 36 h after the injection (Figure 6B-a and b). Similarly, the proteolytic activity in the larvae also gradually increased in response to injections of various doses of ecdysone (Figure 6C-a), whereas proteolytic activity in these larvae was obviously repressed when the inhibitor CLIK148 was added. The highest level of activity was detected 36 h after a 2-µg injection of ecdysone (Figure 6C-b). From these results, it can be inferred that both the expression and activity levels of Har-CL protein can be up-regulated by exposure to ecdysone.

### Ecdysone and Relish are required for regulating Har-CL expression

**Figure 7:**
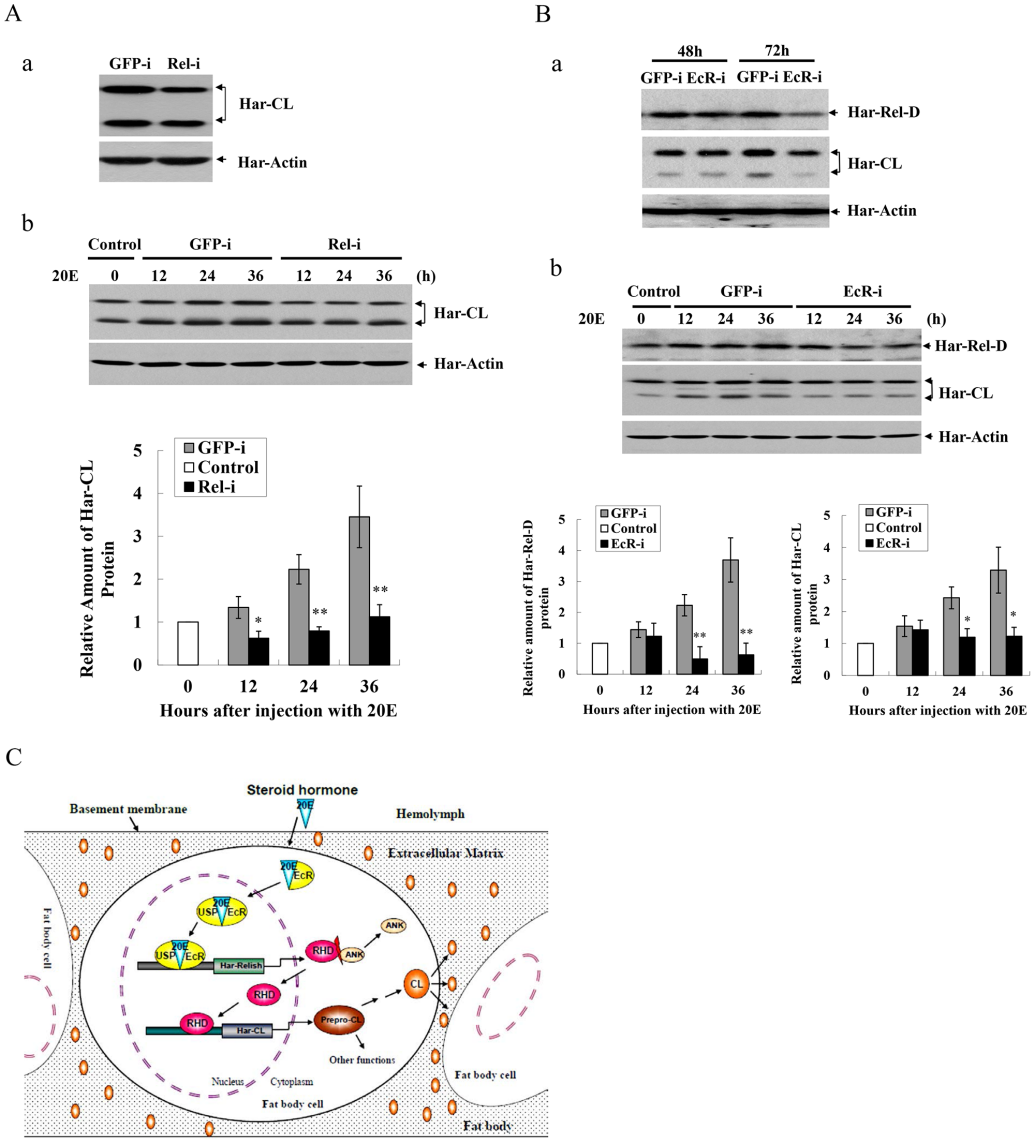
RNAi and a schematic hypothesis for Har-CL gene regulation. (A) Knocking down Har-Relish and western blot analysis by RNAi. (a) Expression of Har-CL protein after knocking down Har-Relish. Day 4 of the sixth instar larvae were injected with Har-Relish dsRNA (Rel-i) or GFP dsRNA (GFP-i), and protein was extracted from fat body after a 48-h incubation period and used for western blotting. (b) Western blotting for Har-CL after injection of ecdysone (20E). Day 4 of the sixth instar larvae were injected with Har-Relish dsRNA (Rel-i) or GFP dsRNA (GFP-i) for 48 h, and then 2 µg of 20E was injected. Protein was extracted from the larval fat body with a 12-h interval after injection and used for western blot. The protein bands (propeptide and mature peptide) were quantified and normalized to the level of Har-actin. (B) Knocking down Har-EcR by RNAi and western blot analysis. (a) Expressions of Har-Relish and Har-CL proteins after knocking down Har-EcR. Day 4 of the sixth instar larvae were injected with Har-EcR dsRNA (EcR-i) or GFP dsRNA (GFP-i), and protein for western blot was extracted from fat body 48 and 72 h after injection. (b) Western blot for Har-Relish and Har-CL by injection of 20E. Day 4 of the sixth instar larvae were injected with Har-EcR dsRNA (EcR-i) or GFP dsRNA (GFP-i) for 48 h, and then 2 µg of 20E was injected. Protein for western blot was extracted from fat body with a 12-h interval. The protein bands from three repeats were quantified and normalized to the level of Har-actin. Error bars represent S.D.s, * indicates a *p* value of <0.05, and ** indicates a *p* value <0.01; *p* values were determined using paired one-sample *t*-tests. (C) Schematic description of a hypothesis on the regulatory mechanism of Har-CL gene expression in the fat body to degrade extracellular matrix for the dissociation of the fat body cells.

To determine whether ecdysone can up-regulate Har-CL expression and activity via the activation Har-Relish, we performed RNAi experiments to suppress the expression of *Har-Relish* in HzAM1 cells or the sixth instar larvae. In the HzAM1 cell line, exposure to ecdysone is capable of up-regulating the expression of both Har-Relish and Har-CL mRNAs when the cells are transfected with dsRNAs that encode a GFP. However, the expression levels of both Har-Relish and Har-CL mRNAs clearly decreased when *Har-Relish* was knocked down by transfection with Relish dsRNA (Figure S7A). Otherwise, the down-regulation of Har-Relish protein in the fat body (Figure S7B-a) can result in a decrease of Har-CL protein (Figure 7A-a). The result showed Relish regulating Har-CL expression again. Two microgram of 20E was then injected into the sixth instar larvae that had been treated with the GFP dsRNA for 48 h. After injection of 20E, the level of Har-CL protein expression in the fat body was elevated between 12 and 36 h after injection (Figure 7A-b). However, in the larvae in which *Relish* expression had been down-regulated via a 48-h treatment with Har-Relish dsRNA, the level of Har-CL protein expression in the fat body did not increase significantly from 12 to 36 h after the injection of 20E.

Finally, we also performed RNAi experiments to suppress *Har-EcR* expression in the sixth instar larvae to block ecdysone pathway, because EcR is ecdysone receptor which binds ecdysone to mediate signaling transduction. Har-EcR mRNA had a decrease significantly from 48 to 72 hours after the injection of Har-EcR dsRNA into the sixth instar larvae (Figure S7B-b). After injection of Har-EcR dsRNA, both Har-CL and Har-Relish proteins in the fat body decreased, especially at 72 h (Figure 7B-a), implying that both of *Har-CL* and *Har-Relish* are regulated by ecdysone signaling pathway. Two microgram of 20E was then injected into the larvae that had been treated with the GFP dsRNA for 48 h, both of Har-Relish and Har-CL proteins in the fat body was elevated from 12 to 36 h after injection with 20E (Figure 7B-b). However, in the larvae in which EcR expression had been down-regulated via a 48-h treatment with Har-EcR dsRNA, the levels of Har-Relish and Har-CL proteins in the fat body did not increase significantly from 12 to 36 h after the injection with 20E. The results clearly show that both of *Har-Relish* and *Har-CL* can response to hormone ecdysone.

## Discussion

Insect fat body is the intermediary metabolism organ and the main source of hemolymph components associated with growth and development. In natural development (nondiapause-type pupa), the fat body must be dissociated into individual fat body cells which are later removed by cell death in *Drosophila* [6]. In Lepidoptera species *H. zea*, a closely related species with *H. armigera* and diapause also is at pupal stage, most of larval fat body cells from the peripheral fat body completely disappear during pupal development, and less fat body cells from the perivisceral fat body reaggregate into adult fat body [26]. The larval fat body cells are likely source of metabolic reserves for pupal-adult development. Inhibition of the fat body dissociation is associated with pharate adult lethality [16]. Thus, the fat body dissociation is an essential developmental event. In contrary, the larval fat body will remain integral in diapause-type pupae, and result in developmental arrest (diapause) towards adult. When diapause is broken, the fat body starts to be dissociated and then pupal-adult development is restarted. Therefore, *H. armigera* pupal diapause is a useful animal model to study the molecular mechanism for tissue remodeling in the fat body.

### Har-CL functions in the dissociation of the larval fat body

In mammalian, lysosomal cysteine proteases, such as cathepsin L, B and K, are synthesized and targeted to the acidic compartments, lysosomes and endosomes, by either the mannose-6-phosphate receptor-dependent pathway [27], [28] or the mannose-6-phosphate receptor-independent pathway [29]. Cysteine proteases are also released to ECM and involved in ECM degradation by “focal contact” [30], [31]. The regulatory process may include: the proteases are moved to plasma membrane and released into ECM by exocytosis; the formation of a localized acidic environment in a zone of contact that excludes the surrounding extracellular milieu and increased expression of vacuole-type H^+^-ATPase [30], [31]. However, it is unknown whether the regulatory mechanism is conserved in insects. Our previous study demonstrated the Har-CL protein sequence, including pro-region and mature enzyme region, possesses considerable sequence homology with human cathepsin (54%) [18], it implies that a similar mechanism for the ECM degradation by cathepsin may exist in insects, although the detailed regulatory and molecular factors require further investigation.

In previous investigation of Har-CL involvement in larval moulting, we found a clear cathepsin activity and expression difference between the whole body and hemolymph of day 0 pupae [18]. We concluded that high cathepsin activity in the whole body may originate from the fat body. To test this hypothesis, we investigated the changes of cathepsin activities in the fat body from the 5^th^ instar larvae to day 0 pupae of diapause- and nondiapause-destined. The results demonstrated that high cathepsin expression in whole body of day 0 pupa is derived from the fat body, and more than 90% of proteolytic activity in the fat body of day 0 pupae was from cathepsin L protease. However, the functional significance of Har-CL in pupal fat body still remains uncertain.

Larval fat body must be degraded at early pupal stage, and then new adult fat body is formed during pupal-adult development. But, the dissociation mechanism for fat body is poorly reported. Previous studies suggested a hypothesis that the dissociation of the fat body is mediated by hemocytes that associate with and degrade the basement membrane of the fat body through release of cathepsin B or aspartyl protease [9]–[11]. However, the fat body dissociation and remodeling in *D. melanogaster* was recently demonstrated to be a hemocyte independent process [16]. More recently, Bond *et al.* [17] showed that βFTZ-F1 was involved in *Drosophila* fat body remodeling through the regulation of the MMP-2 expression. These results showed that the dissociation of the fat body may be caused by an internal factor, but not hemocyte. In this study, we demonstrated that cathepsin L protease, an internal factor, participates in the degradation of the larval fat body at early pupal stage.

Firstly, both Har-CL mRNA and protein expressed heavily in the fat body of day 0 pupa and its activity also is high, coincident with the dissociation of the fat body on day 0. Secondly, immunostaining demonstrated that Har-CL-positive signals in nondiapause pupae moved to the surface from middle of the fat body cells and then released Har-CL from the lysosomes, but Har-CL in diapause-type pupae still remained in lysosomes of the fat body cells and randomly distributed in cytoplasm. Western blot and activity analysis for cathepsin demonstrated that Har-CL of the fat body did not release into hemolymph of day 0 nondiapause pupae. These results suggest that Har-CL in the fat bodies of day 0 nondiapause-type pupae is associated with degradation of the ECM and basement membrane for remodeling new adult fat body. Finally, the inhibitor for Har-CL and dsRNA against Har-CL experiments demonstrated clearly that the fat body dissociation can be repressed significantly by inhibiting cathepsin L activity or knock-down of Har-CL expression. In treated individuals, less dissociated fat body cells are still detected in the hemolymph, implying that cathepsin L in *H. armigera* is the most important enzyme to degrade extracellular matrix for fat body dissociation, and other cathepsin members may also contribute to the fat body dissociation, as cathepsin L-selective inhibitor CLIK148 can inhibit approximately 90% of the activity in the fat body as shown in Figure 1C. Thus, Har-CL, an internal factor from the fat body, does play a crucial role in the dissociation of larval fat body in *H. armigera*.

### Relish is required for the regulation of Har-CL gene expression

In insects, the NF-κB homolog *Dorsal* was first identified as having a role in the regulating the specification of embryonic dorsal-ventral polarity in *D. melanogaster* [32]. Later, Relish and Dorsal/Dif were identified in *D. melanogaster*, *B. mori*, and other species, but these genes generally have functions in regulating the transcription of antimicrobial peptide genes that support innate immunity [33]–[36]. In the present study, we identified *Har-CL* promoter region which has an NF-κB homolog-binding site, and the nuclear extract protein HCLMBP-2 could bind to the NF-κB-binding site. The result strongly implied that HCLMBP-2 may be a member of the NF-κB protein family. The Dorsal and Relish cDNAs from *H. armigera* were then cloned, and we found that Har-Relish was able to bind to the promoter of Har-CL gene and regulate its activity, whereas Dorsal was unable to modulate the activity of Har-CL gene. Our results suggest that the nuclear protein HCLMBP-2 is Har-Relish, which is an NF-κB homolog based on the following four observations: (i) the interaction between HCLMBP-2 and its specific probe LP1 could be competitively disrupted by the unlabeled ATTκB probe, which is a short probe that has previously been reported to bind NF-κB in *B. mori* [22], [23]; (ii) complexes of LP1 and HCLMBP-2 had the same mobility as complexes of LP1 and *in vitro-*translated Har-Rel-D; (iii) both Har-Relish and Har-Rel-D can effectively activate *Har-CL* promoter in co-transfection assays, whereas a deletion in the NF-κB-binding site can significantly reduce the activity of *Har-CL* promoter; (iv) Relish can bind to *Har-CL* promoter *in vivo*.

Members of NF-κB family have a key role in the inflammatory and immune responses by inducing expressions of cytokines, chemokines, and their receptors [37]. NF-κB also has other roles except transcription regulation, such as reducing expression of transcription factor MyoD by disturbing mRNA stability, and affecting tumor promoting or suppressing by regulating oncogenes cyclin D1, c-Myc, p53 [38]. In insects, all Relishs are related to innate immune response [39]. Interestingly, Relish in *H. armigera* can bind to the promoter of Har-CL gene and activate *Har-CL* expression to regulate the dissociation of insect fat body.

From Northern blot, Har-Relish was highly expressed in the fat body. In *Drosophila*, the fat body has important functions in growth and metamorphic through TSC/TOR or insulin signaling pathways [8], [40], [41]. Therefore, fat body plays an essential role in insect development and metamorphosis by activating certain gene expressions, such as *Har-CL* and *Har-Relish*. In this study, both *Har-CL* and *Har-Relish* are highly expressed in the fat body, and have the same expression pattern during larval-pupal development. The result implies a closely relationship between Har-CL and Har-Relish. When down-regulation of Har-Relish expression by RNAi, both Har-CL mRNA and protein decreased significantly. Thus, Har-Relish expressed in the fat body contributes to the dissociation of the fat body at early pupal stage by regulating *Har-CL* expression.

### Har-Relish can respond to ecdysone signaling

Ecdysone is an important hormone to regulate development and metamorphosis in insects. Likewise, ecdysone can genetically regulate immune response genes in *D. melanogaster* and *B. mori*, implying that ecdysone may regulate the expression of NF-κB [42], [43]. Previous studies have demonstrated that Har-CL gene expression in *H. armigera* larvae responded to exposure to ecdysone, but the underlying mechanism that mediates Har-CL gene regulation is unknown [18], [44]. In the present study, the expression of *Har-Relish* in the 6^th^ instar larvae increased significantly during the 12 h following an injection of ecdysone; this response to ecdysone was more rapid than the response of *Har-CL*, the expression of which only increased significantly 24–36 h after ecdysone injection. Har-Relish did not increase significantly during the 12–36 h following the injection of distilled water, which was used as a control (data not shown). This finding is contrary to the reports that in *Drosophila*, injection with nothing could temporarily increase the expression of *Relish*, but the change in the level of *Relish* expression was not significant after 6 h [45], [46]. If *Har-Relish* expression had been knocked down as a result of RNAi, the level of *Har-CL* expression was significantly decreased until 36 h after treatment with ecdysone. This result shows that there is a cascade of gene expression in which ecdysone regulates the expression of *Har-Relish*, after which Har-Relish activates Har-CL gene expression both *in vivo* and *in vitro*. Thus, one mechanism for the action of ecdysone is that it is released into hemolymph from the prothoracic glands as an up-stream signal, after which it binds to its receptors EcR and USP in the fat body, and finally regulates the expression of Har-CL gene by activating the transcription factor Har-Relish. This hormone (ecdysone)-transcription factor (Relish)-target gene (cathepsin) regulatory pathway for the dissociation of larval fat body is shown in Figure 7C.

Recently, we detected ecdysone titers in diapause- and nondiapause-destined pupae, showed that ecdysone level in diapause-type pupae is much lower than in nondiapause-type ones [47]. It is well known that if injection of ecdysone into diapausing pupae to elevate ecdysone level, the fat body will start dissociation and restart pupal-adult development. The results showed that high level of ecdysone promotes pupal-adult development with Har-CL release from the fat body and low ecdysone induces pupal diapause without Har-CL release, implying that ecdysone not only regulates Har-CL expression during larval development, as well as mediates Har-CL release after pupation.

In addition, we found evidence that Har-Dorsal, another NF-κB homologue that we had cloned in a previous experiment, is a transcription factor that belongs to the NF-κB family. Har-Dorsal contains an RHD, a proline-rich region, and a leucine zipper, and, as we found in a preliminary experiment, it was also expressed in the fat body (data not shown). However, Har-Dorsal was unable to bind to Har-CL promoter to regulate its transcriptional activity. In *D. melanogaster* and *B. mori*, Relish regulates some genes that cannot be regulated by Dorsal, and Dorsal regulates some genes that cannot be regulated by Relish [22], [23], [34]. Additional future experiments are needed to clarify the precise function of the *Dorsal* in *H. armigera*.

The activity assay of *Har-CL* promoter showed that one segment (from −308 to −287) resulted in a strong transcriptional activity, and two segments (from −522 to −345 and from −1554 to −1292) had suppressive activities (Figure 4A). Therefore, we assume that a specific transcription factor may bind to the activating region of the promoter. A homology search was used to identify an E-box-binding site at which the transcription factor Myc is able to binds. It will be interesting to conduct further investigations of whether Myc regulates Har-CL gene because Myc is an important regulator of cell growth and proliferation. Some inhibitors may bind to sites in the suppression regions of the promoter and may then down-regulate Har-CL transcription. However, in conducting a homology search, we were unable to find a candidate element that was capable of binding to the suppression regions. Further tests that aim to identify inhibitory binding elements require EMSA experiments that utilize the incubation of the two suppression segments and with nuclear protein extracts.

In promoter activity analysis, HLP2Δ promoter, which deleted the −287∼−308 region, but contained the −345∼−308 region, still obtained a low activity (Figure 4B). We speculate that loss of −287∼−308 region may affect transcription factor binding to −345∼−308 region, and result in low luciferase activity, as transcription factor binding or no will affect adjacent other transcription factor binding as reported previously [48], implying that the interactions by transcription factors in the promoter are important for activating the transcription of Har-CL gene.

## Materials and Methods

### Insects

*H. armigera* larvae were reared on an artificial diet at 25±1°C, with a light-dark cycle of L14∶D10 (nondiapause type) and 18±1°C, with a photoperiod of L10∶D14 (diapause type) [49]. In nondiapause type, development for the sixth instar larvae is approximately 6 days, and 13 days in diapause type. To accurately compare gene expression at the same time, *H. armigera* larvae were also reared at 20°C, with L14∶D10 (nondiapause type) or L10∶D14 (diapause type) to synchronize developmental time.

### Proteolytic activity assay

Proteolytic activity was assayed using a specific substrate of cathepsin B and L, Z-Phe-Arg-MCA (Z-F-R-MCA, Sigma). Protein extracts (10 µg) or hemolymph (2 µl) were preincubated at 37°C for 10 min in 80 µl of Na_2_HPO_4_-citrate buffer (pH 4.4), containing 1.25 mM EDTA and 10 mM cysteine to activate the cysteine proteases, and then incubated for another 10 min after 10 µl 1 mM Z-F-R-MCA was added. After incubation, 100 µl 10% SDS and 2 ml Tris-HCl buffer (pH 9.0) were added to terminate the reaction. The fluorescence of liberated aminomethylcourmarin (AMC) molecules was measured by a fluorometer at an excitation of 370 nm and an emission of 460 nm. To inhibit proteolytic activity, 1 and 10 µM of the cysteine protease inhibitor E-64 (Sigma), the cathepsin B-selective inhibitor CA074 (Sigma) or the cathepsin L-selective inhibitor CLIK148 [50] were used. The inhibitors were first dissolved in DMSO to make a 20-mM stock solution, stored at −20°C, and prior to assay diluted with ultra-pure water. Thus, cathepsin L activity was deduced from the total activities and cathepsin B activity.

### Western blot analysis

For Western blot analysis, protein extracts (80 µg for Har-Relish, 20 µg for Har-CL of the fat body sample, and 30 µg for Har-CL of the hemolymph sample) were separated on 12% SDS-PAGE and transferred on to a PVDF membrane (Hybond-P, Amersham). Non-specific binding was blocked using a 5% milk without fat solution, and Har-Relish protein was detected with the Novex Chemiluminescent Substrate (Invitrogen, Carlsbad, USA) in three separate experiments independently.

Cathepsin L is synthesized as precursors, and activated by proteolytic removal at the N-terminal of propeptide [51]. Both propeptide and mature protein represented protein level of cathepsin L as reported in Liu et al. [18] and Jean et al.[52], so we quantified from the two bands (propeptide and mature peptide) as described in Tang et al.[53], and normalized to the level of Har-Actin.

### Whole-mount immunocytochemistry

*H. armigera* larvae were reared at 20°C, with L14∶D10 (nondiapause type) or L10∶D14 (diapause type) to synchronize developmental time. Fat body was dissected in 0.75% NaCl, fixed in PBT (PBS+0.3% Triton-X100) containing 3.7% formaldehyde for 2 h at room temperature, and extensively washed in PBT. Tissues were then blocked for 30 min in PBT containing 10% normal goat serum. Anti-Har-CL antibody (1∶500) was incubated overnight at 4°C and secondary antibody (goat anti-rabbit Alexa flour 488, 1∶1000) for 1 h at room temperature. Nucleus was stained with 5 µg/ml Hoechst 33342. Tissues were mounted in mounting medium, and the fluorescence images were acquired using a confocal laser scanning microscope (TCS-SP5, Leica). In the control experiments, the primary antibodies were replaced by pre-immunized rabbit serum.

### Transient transfection and luciferase assay

HzAM1 cells were seeded in 96-well cell culture plates, cultured at 27°C with Grace's Insect Cell Culture Medium (GIBCO) supplemented with 10% fetal bovine serum overnight to let cells grow to *log* phase. Then cells were cultured without antibiotics or serum. Transfection was performed with the Cellfectin II Reagent (Invitrogen) as described in the user manual. The recombinant plasmid (200 ng) and 0.6 µl Cellfectin II were mixed in 50 µl Grace's Insect Medium without antibiotics or serum. After incubation at room temperature for 20 min, the DNA-lipid mixture was added into the cell culture medium gently. After 5–7 h, the transfection mix was replaced by complete medium with 10% fetal bovine serum. Each transfection was repeated three times. After incubation for 48 h, the cells were washed with cold PBS and harvested in Passive Lysis Buffer (Promega). Luciferase activity was assayed using the Dual-Luciferase Reporter Assay System (Promega) according to the manual by a microplate luminometer MikroWin2000 (Mikrotex) in three separate experiments independently.

### *In vitro* translation and EMSA assay

The pET28-Har-Rel-D plasmid was used as a template for *in vitro* translation in the TNT Quick Coupled Transcription/Translation System (Promega). The reaction system contained pET28-Har-Rel-D plasmid (1 µg), TNT T7 Quick Master Mix, 1 mM methionine, and was incubated at 30°C for 1 h. The translation product was used for the EMSA assay.

Nuclear protein extracts were prepared from *H. armigera* fat body according to the procedures described in Zhang et al. [48]. The probes used in the experiment were prepared through PCR, then digested with *Eco*R I or by annealing two overlapping oligonucleotides. The gaps in the probes produced by annealing oligonucleotides with partial overlapping were filled using a Klenow fragment (TaKaRa) at 37°C for 30 min in the presence of [α-^32^P]-dATP, dCTP, dGTP and dTTP for labeling probes. Competitive probes were produced by the same method, but dATP was used instead of [α-^32^P]-dATP. DNA-binding reactions were performed by incubating nuclear extracts (5–10 µg) at 27°C for 30 min with ^32^P-labelled double-stranded DNA (10 000 c.p.m.) in binding reaction buffer [10 mM Hepes-K^+^ (pH 7.9), 10% glycerol, 50 mM KCl, 4 mM MgCl_2_, 1 mM DTT, 0.5 mg/ml BSA, 0.1 mM PMSF and 1 µg of poly(dI/dC) (Sigma)]. Samples were resolved on 5% (w/v) non-denaturing polyacrylamide gel in 1×TBE at 150 V. After electrophoresis, the gel was dried and subjected to autoradiography using an intensifying screen at −80°C for 16 h. For competition experiments, a 100-fold excess of unlabeled probe was pre-incubated with the nuclear protein extracts at 27°C for 10 min and then subjected to the procedures as mentioned above.

### RNA interference

Using a micro-injector (Hamilton Company), Har-CL (15 µg) dsRNA was injected into larvae (the last day of the sixth instar), approximately 18 h before pupation, and GFP dsRNA was injected as a control. By our preliminary experiments, 15 µg of Har-CL dsRNA was injected into larvae, over 80% larvae could normally molt into pupae. DsRNAs of Har-EcR (25 µg) or Har-Relish (15 µg) were injected into day 4 of the sixth instar larvae, and the GFP dsRNA was used as a control. Forty-eight hours after injection, 2 µg of 20E was injected into the larvae again.

### Insect treatments, protein preparation, polyclonal antibody generation, Northern blot, competitive RT–PCR and Southern blot analysis, genome walking, cloning of Relish and Dorsal cDNAs, construction of reporter gene and deletion mutagenesis, construction of the overexpression system, chromatin immunoprecipitation (ChIP) assay, and DsRNA generation

Details of these methods can be found in the Supporting Materials and Methods section of Text S1 and Table S1.

### Statistical analysis

All data were statistically analyzed by independent sample *t*-test. Asterisks indicate significant differences (*, *p*<0.05; **, *p*<0.01).

## Supporting Information

Figure S1 Immunohistochemical analysis of Har-CL in fat body. Har-CL positive signals (green) are localized in the fat body of nondiapause- and diapause-destined individuals (bar = 50 µm), and the nucleus were stained with blue. The mock was treated with preimmunized rabbit serum as the control. Fat body from day 5 (6L5, feeding stage) (A) and day 9 (6L9, pre-pupal stage) (B) of the 6^th^ instar larvae; Fat body from 0 h (P0h) (C) and 24 h (P24h) (D) after pupation. (E) Fat body of day 20 diapausing pupa (P20D).
(TIF)

Figure S2 Western blot and proteolytic analysis of Har-CL. (A) Proteins from hemolymph (He, 35 µg) and fat body (FB, 20 µg) of day 0 nondiapause pupae were separated on SDS-PAGE for immunoblot analysis using Har-CL polyclonal antibodies. (B) Proteolytic activities from hemolymph (He) and fat body (FB) of day 0 nondiapause pupae were measured as described in Figure 1. Error bars represent S.D.s that were obtained from three independent experiments.
(TIF)

Figure S3 Fat body cells in hemolymph of day 0 pupa. (A) Hemolymph was collected 6, 12, 18, and 24 h after pupation, and dripped onto the glass slide, immediately photographed and observed by light microscope. Scale bar: 200 µm. (B) Each individual were collected hemolymph 2.5 µl three times to count the cells. Error bars represent S.D.s that were obtained from ten independent experiments.
(TIF)

Figure S4 Structural characterization of the promoter region of Har-CL gene. A nucleotide sequence that is upstream of Har-CL gene in the 5′ direction is cloned and sequenced from *H. armigera* genome. A predicted transcriptional start site (+1) is indicated with an arrow. Potential consensus sequences for regulatory element and transcription factor-binding sites are underlined.
(TIF)

Figure S5 Characteristics of Har-Relish and Har-Dorsal cDNAs. (A) Deduced amino acid sequences from Har-Relish cDNA (a) and Har-Dorsal cDNA (b). Bold letters indicate the Relish homology domain (RHD). The box indicates the death domain. Five sequences of Ankyrin repeat (ANK) are underlined. Closed arrowhead denotes the caspase cleavage site. (B) Sequence alignment of Har-Relish (a) or Har-Dorsal (b) domains with other insects.
(TIF)

Figure S6 Tissue distribution and developmental expression of Har-Relish gene. (A) Northern blot analysis. Total RNA (35 µg) was extracted from the brain (Br), trachea (Tr), midgut (Mg), cuticle and epidermis (Ce), fat body (Fb), and Malpighian tubule (Mt) of the sixth instar, day 3 larvae were separated on a 1.2% formaldehyde-agarose gel and were hybridized using a radiolabeled Har-Relish cDNA probe; 18S rRNA was used as a loading control. (B) Semi-quantitative RT-PCR. Har-Relish mRNAs were isolated from the fat bodies of sixth instar larvae of various ages and from new pupae, and a truncated Har-Relish cDNA is used as a control (T-Har-Relish). (C) Western blotting analysis. Har-Relish proteins from the fat bodies of sixth instar larvae of various ages and from new pupae were detected, and Har-Actin is used as a control.
(TIF)

Figure S7 RNAi knockdown efficiency. (A) Knock down of Har-Relish in HzAm1 cells. HzAM1 cells were treated with Har-Relish dsRNA (Rel-i) or GFP dsRNA (GFP-i) for 48 h, then ecdysone (20E) was added into HzAM1 cells. Total RNA was extracted 24 h after addition of 20E, and RT-PCR was performed. The numbers 1, 2 and 3 represent three experiments independently. (B) RNAi knockdown efficiency in the fat body injected with Har-Relish or Har-EcR dsRNAs. (a) Expression levels of Har-Relish protein 48 h after injection of Har-Relish dsRNA. Har-Actin was as a control. (b) Changes of Har-EcR transcript levels. Total RNA was extracted 48 h and 72 h after injection of Har-EcR dsRNA, and RT-PCR was performed to amplify the RNA extracts. RPL32 was as a control. The numbers 1, 2 and 3 represent three independent experiments.
(TIF)

Table S1 Oligonucleotides used for plasmid constructed or EMSA.
(DOCX)

Text S1 Supporting information on Materials and Methods.
(DOCX)
