# Supplementary figures and images for: A Regulatory Pathway, Ecdysone-Transcription Factor Relish-Cathepsin L, Is Involved in Insect Fat Body Dissociation
Source: PLoS Genet. 2013 Feb 14;9(2):e1003273. doi: 10.1371/journal.pgen.1003273 (PMC3573115; doi:10.1371/journal.pgen.1003273)

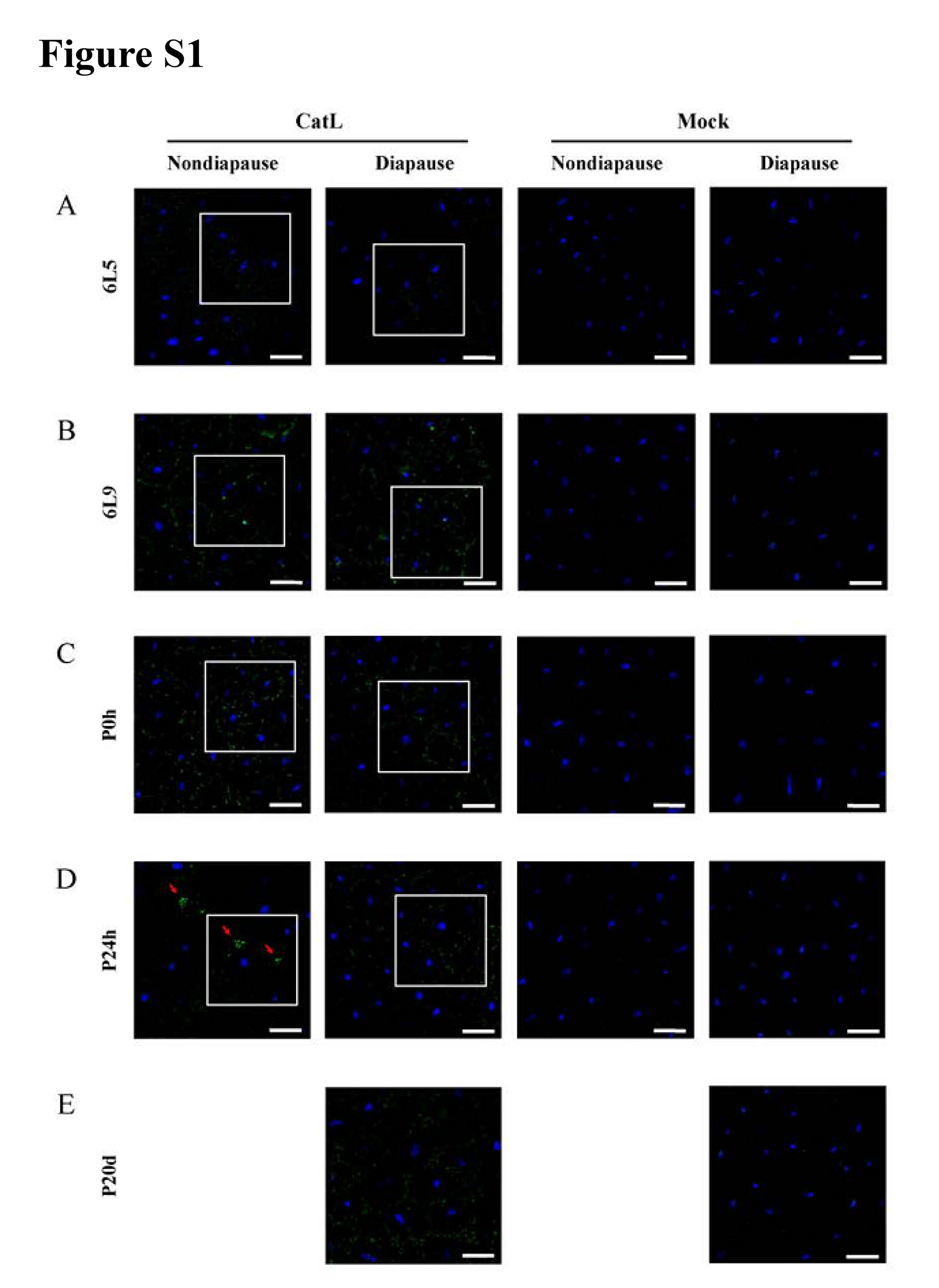

Supplement: Figure S1 — Immunohistochemical analysis of Har-CL in fat body. Har-CL positive signals (green) are localized in the fat body of nondiapause- and diapause-destined individuals (bar = 50 µm), and the nucleus were stained with blue. The mock was treated with preimmunized rabbit serum as the control. Fat body from day 5 (6L5, feeding stage) (A) and day 9 (6L9, pre-pupal stage) (B) of the 6th instar larvae; Fat body from 0 h (P0h) (C) and 24 h (P24h) (D) after pupation. (E) Fat body of day 20 diapausing pupa (P20D). (TIF) [file pgen.1003273.s001.tif]

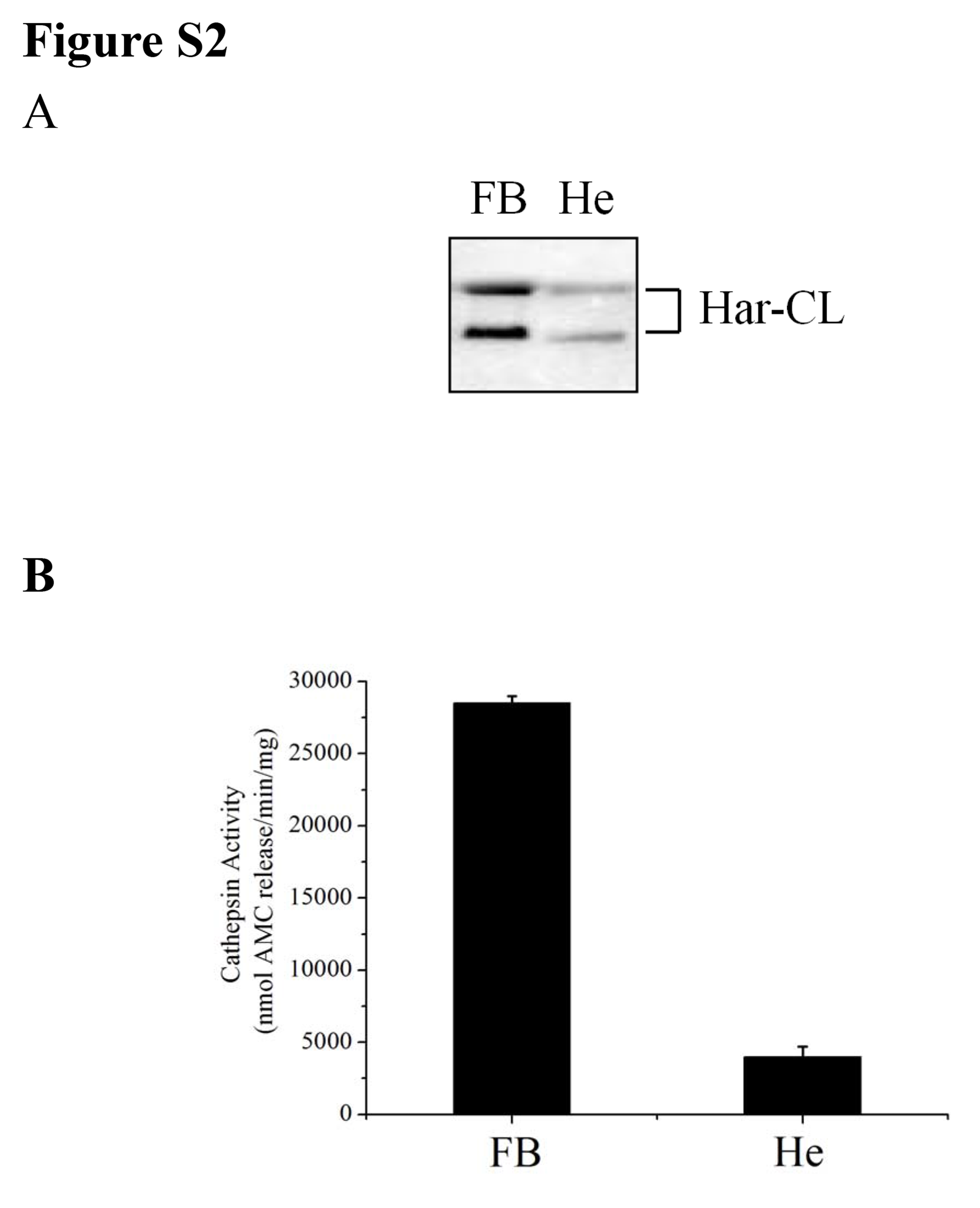

Supplement: Figure S2 — Western blot and proteolytic analysis of Har-CL. (A) Proteins from hemolymph (He, 35 µg) and fat body (FB, 20 µg) of day 0 nondiapause pupae were separated on SDS-PAGE for immunoblot analysis using Har-CL polyclonal antibodies. (B) Proteolytic activities from hemolymph (He) and fat body (FB) of day 0 nondiapause pupae were measured as described in Figure 1. Error bars represent S.D.s that were obtained from three independent experiments. (TIF) [file pgen.1003273.s002.tif]

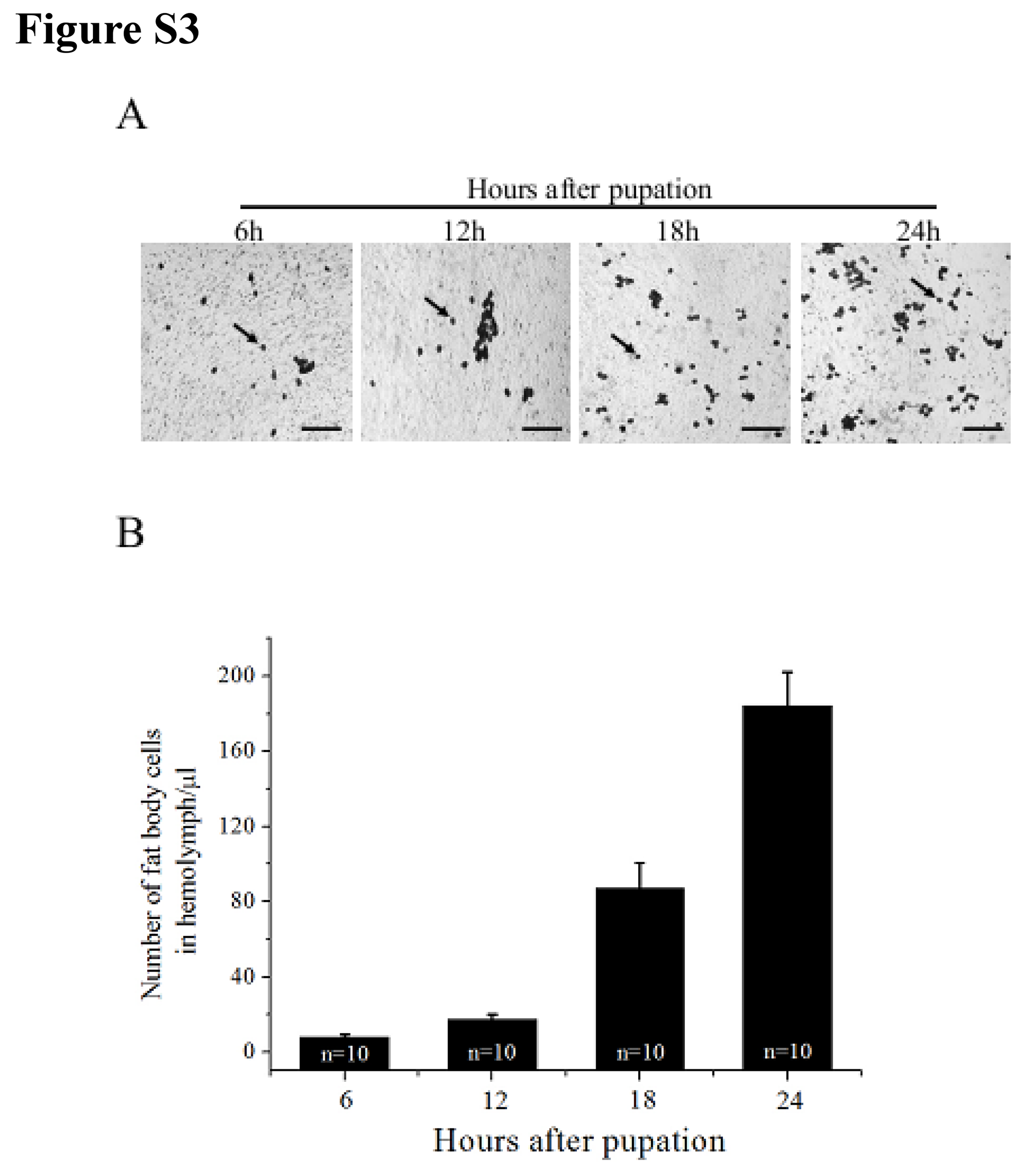

Supplement: Figure S3 — Fat body cells in hemolymph of day 0 pupa. (A) Hemolymph was collected 6, 12, 18, and 24 h after pupation, and dripped onto the glass slide, immediately photographed and observed by light microscope. Scale bar: 200 µm. (B) Each individual were collected hemolymph 2.5 µl three times to count the cells. Error bars represent S.D.s that were obtained from ten independent experiments. (TIF) [file pgen.1003273.s003.tif]

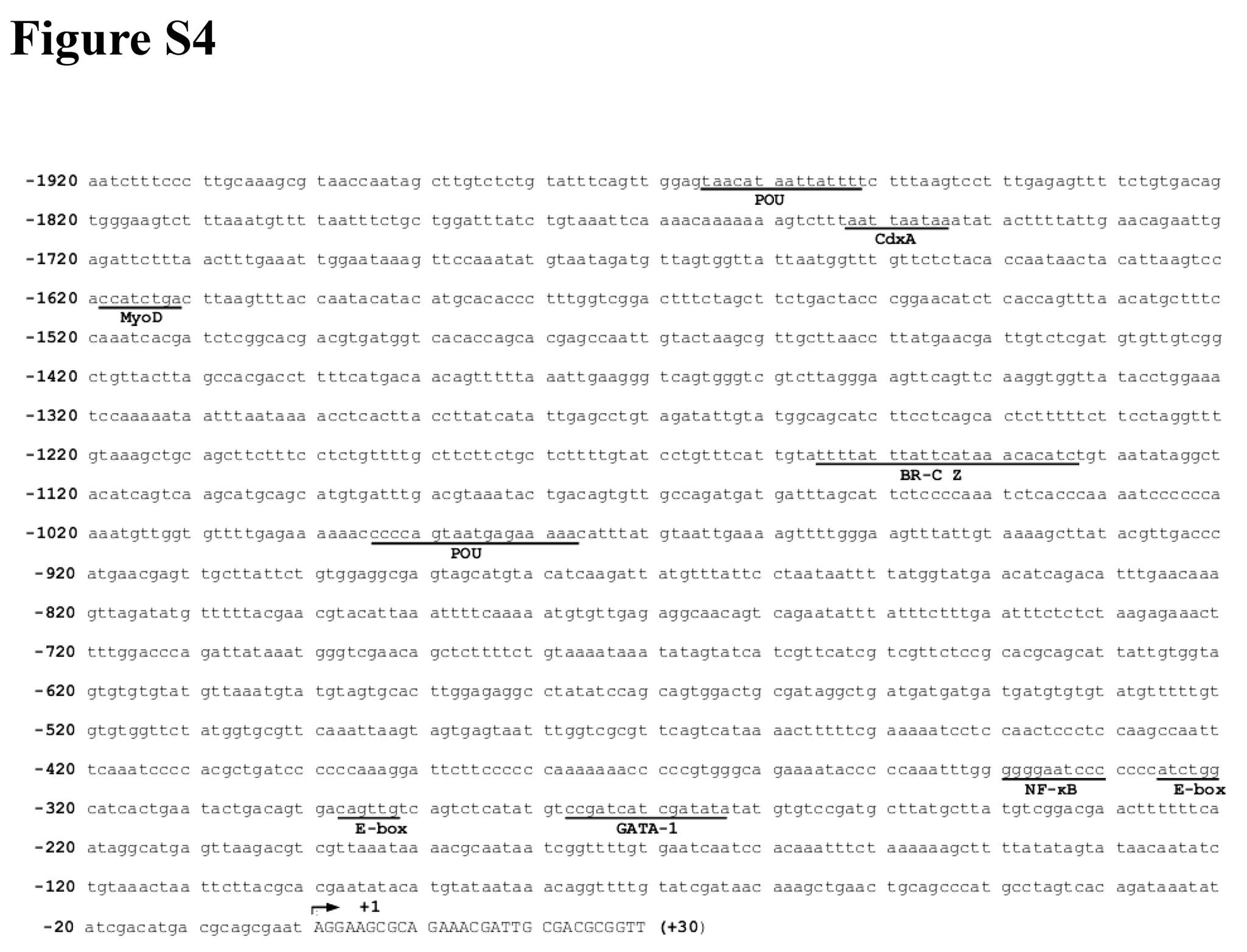

Supplement: Figure S4 — Structural characterization of the promoter region of Har-CL gene. A nucleotide sequence that is upstream of Har-CL gene in the 5′ direction is cloned and sequenced from H. armigera genome. A predicted transcriptional start site (+1) is indicated with an arrow. Potential consensus sequences for regulatory element and transcription factor-binding sites are underlined. (TIF) [file pgen.1003273.s004.tif]

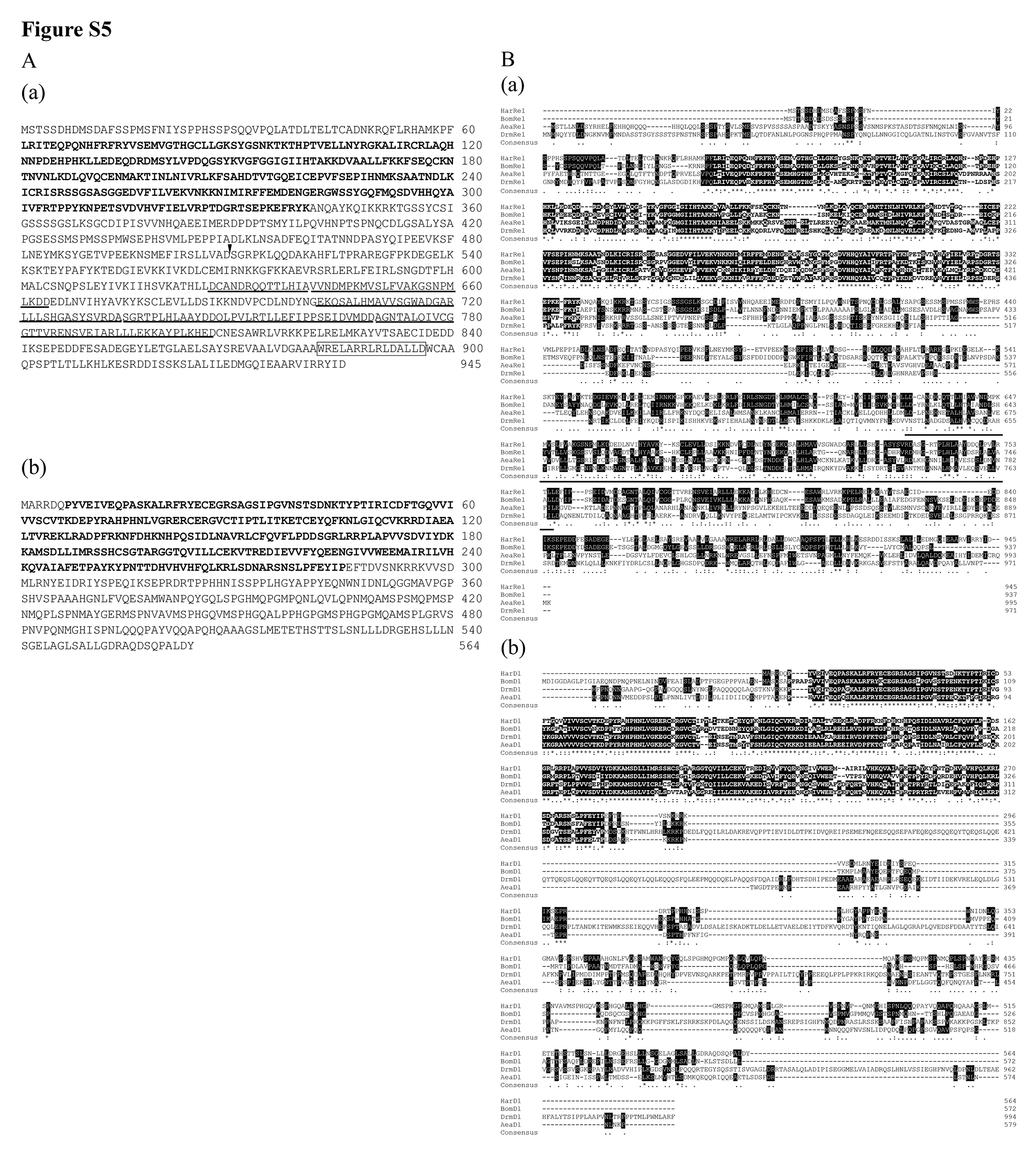

Supplement: Figure S5 — Characteristics of Har-Relish and Har-Dorsal cDNAs. (A) Deduced amino acid sequences from Har-Relish cDNA (a) and Har-Dorsal cDNA (b). Bold letters indicate the Relish homology domain (RHD). The box indicates the death domain. Five sequences of Ankyrin repeat (ANK) are underlined. Closed arrowhead denotes the caspase cleavage site. (B) Sequence alignment of Har-Relish (a) or Har-Dorsal (b) domains with other insects. (TIF) [file pgen.1003273.s005.tif]

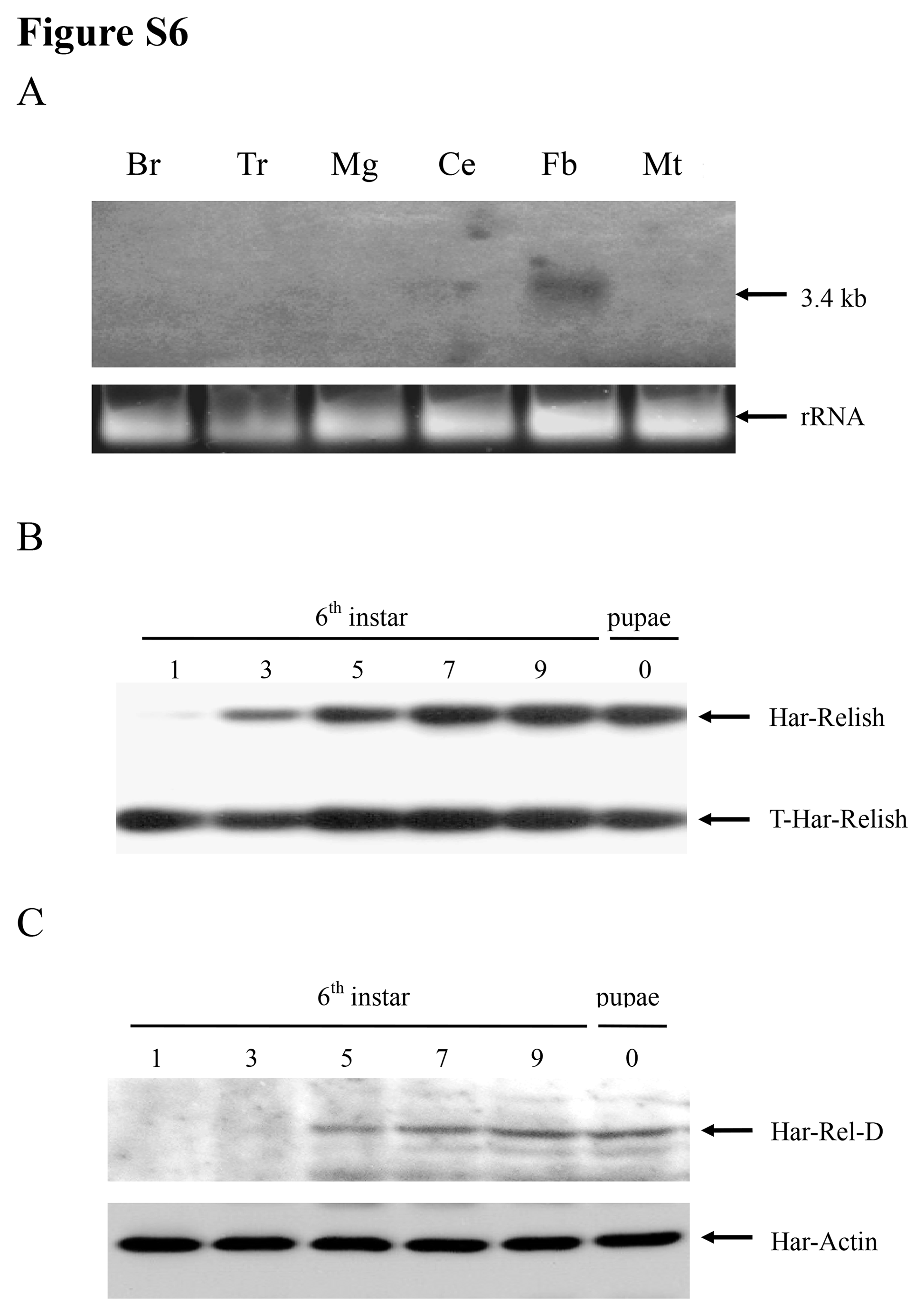

Supplement: Figure S6 — Tissue distribution and developmental expression of Har-Relish gene. (A) Northern blot analysis. Total RNA (35 µg) was extracted from the brain (Br), trachea (Tr), midgut (Mg), cuticle and epidermis (Ce), fat body (Fb), and Malpighian tubule (Mt) of the sixth instar, day 3 larvae were separated on a 1.2% formaldehyde-agarose gel and were hybridized using a radiolabeled Har-Relish cDNA probe; 18S rRNA was used as a loading control. (B) Semi-quantitative RT-PCR. Har-Relish mRNAs were isolated from the fat bodies of sixth instar larvae of various ages and from new pupae, and a truncated Har-Relish cDNA is used as a control (T-Har-Relish). (C) Western blotting analysis. Har-Relish proteins from the fat bodies of sixth instar larvae of various ages and from new pupae were detected, and Har-Actin is used as a control. (TIF) [file pgen.1003273.s006.tif]

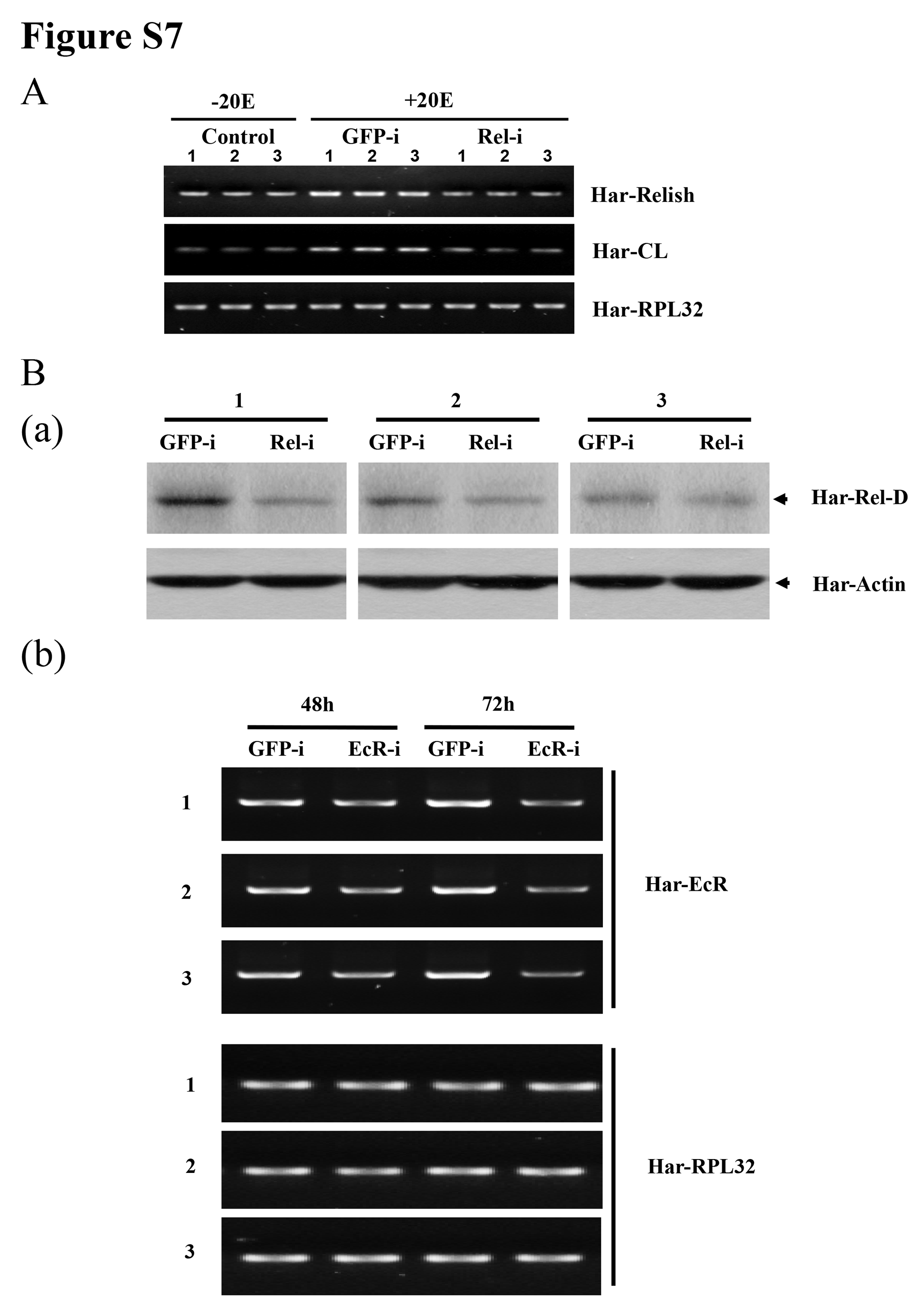

Supplement: Figure S7 — RNAi knockdown efficiency. (A) Knock down of Har-Relish in HzAm1 cells. HzAM1 cells were treated with Har-Relish dsRNA (Rel-i) or GFP dsRNA (GFP-i) for 48 h, then ecdysone (20E) was added into HzAM1 cells. Total RNA was extracted 24 h after addition of 20E, and RT-PCR was performed. The numbers 1, 2 and 3 represent three experiments independently. (B) RNAi knockdown efficiency in the fat body injected with Har-Relish or Har-EcR dsRNAs. (a) Expression levels of Har-Relish protein 48 h after injection of Har-Relish dsRNA. Har-Actin was as a control. (b) Changes of Har-EcR transcript levels. Total RNA was extracted 48 h and 72 h after injection of Har-EcR dsRNA, and RT-PCR was performed to amplify the RNA extracts. RPL32 was as a control. The numbers 1, 2 and 3 represent three independent experiments. (TIF) [file pgen.1003273.s007.tif]
