# Supplementary material for: A Regulatory Pathway, Ecdysone-Transcription Factor Relish-Cathepsin L, Is Involved in Insect Fat Body Dissociation
Source: PLoS Genet. 2013 Feb 14;9(2):e1003273. doi: 10.1371/journal.pgen.1003273 (PMC3573115; doi:10.1371/journal.pgen.1003273)
Supplement: Table S1 — Oligonucleotides used for plasmid constructed or EMSA. (DOCX) [file pgen.1003273.s008.docx]

| **Table S1** | |
| --- | --- |
| **Oligonucleotides used for plasmid constructed or EMSA** | |
| (1) Primers for stepwise deletion plasmid constructs | |
| Conmmon R | GCTAGC AACCGCGTCGCAATCGTTTC |
| HCLPF1 | GGTACC CTTGCAAAGCG‍TAACC |
| HCLPF2 | GGTACC TGTGACAG‍TGGGAAGTC |
| HCLPF3 | GGTACC TACCCGGAACATCTCACCAG |
| HCLPF4 | GGTACC TACCTTATCATATTGAGCC |
| HCLPF5 | GGTACC CCGCACGCAGCATTATTGTG |
| HCLPF6 | GGTACC GTGTGTGGTTCTATGGTGC |
| HCLPF7 | GGTACC TTTGGGGGGAATCCCCCCCATC |
| HCLPF8 | GGTACC CCGATGCTTATGCTTATGTC |
| *(The Nhe I and Kpn I site underlined in primers)* | |
|  |  |
| (2) Primers for deletion mutant | |
| HLP1muR | TTTGGGGGTATTTTCTGCC |
| HLP1muF | CTGACAGT‍GACAGTTGTCAG |
| HLP2muR | TATTCAGTGATGCCAGATG |
| HLP2muF | CTCATATGTCCGATCATCG |
| HLP3muR | ACTGACAACTGTCACTGTC |
| HLP3muF | CCGATGCTTATGCTTATGC |
| HCLpMR | TTTGGGGGTATTTTCTGCC |
| HCLpMF | CTGGCATCACTGAATACTG |
|  |  |
| (3) Primers for probe generation used for EMSA | |
| LPF | GAATTC TTTGGGGGGAATCCCCCCCATC |
| LPR | GAATTC ACACATATATATCGATGATC |
| *(The EcoR I site underlined in primers)* | |
|  |  |
| (4) Oligonucleotides for EMSA | |
| LP1 | TTTGGGGGGAATCCCCCCCATCTGGCATCACTGAATA |
| Em | TTTGGGGGGAATCCCCCCACGAGTTGATCACTGAATA |
| Nm | TTTGGGGAAGGCCCTCCCCATCTGGCATCACTGAATA |
| ATTкB | TCGATATTAGGGGGAATAATTCTTATCATC |
| nS | TTAAGATTATTTACGCAACCGCATTGCAAAATAAAAC |
| *(The mutant site underlined in oligonucleotides)* | |
|  |  |
