# Supplementary material for: A Regulatory Pathway, Ecdysone-Transcription Factor Relish-Cathepsin L, Is Involved in Insect Fat Body Dissociation
Source: PLoS Genet. 2013 Feb 14;9(2):e1003273. doi: 10.1371/journal.pgen.1003273 (PMC3573115; doi:10.1371/journal.pgen.1003273)
Supplement: Text S1 — Supporting information on Materials and Methods. (DOCX) [file pgen.1003273.s009.docx]

**Text S1**

**MATERIALS AND METHODS**

**Insect treatments**

Five microlitres of 20-hydroxyecdysone (20E, Sigma) was injected into the sixth instar larvae at the thoracic region using a fine glass capillary, and the same concentration of ethanol as control. All tissues were dissected carefully in insect saline including 0.75% NaCl and immediately stored at -80 °C until use.

New pupae (approximately 2 h after pupation) were collected and injected with cathepsin L-selective inhibitor CLIK148 (200 µM, 5µl), or solvent (1% DMSO, 5µl). These injected individuals could develop towards pupae or adults. Hemolymph was collected 22 h after injection, and dripped onto the glass slide, immediately quantified the number of fat body cells with a light microscope (Olympus, DP71).

**Protein preparation**

Protein extracts from fat body were prepared according to Uchida et al. [1] with minor modifications. Briefly, tissues were homogenized in ice-cold extraction buffer (PBS, pH 7.0, 4 mM EDTA, 0.2% Triton X-100). Extract supernatants were collected by centrifugation at 12 000 *g* for 15 min at 4 °C for both proteolytic activity assay and Western blot analysis.

Hemolymph was collected in a tube on ice and centrifuged at 1000 *g* to remove hemocytes, and the supernatant (35 μg protein) was used for Western blot analysis in figure S2.

**Polyclonal antibody generation**

Har-Relish ORF was amplified with two primers RXF and RXR, the PCR product was then purified and digested with *Bam*H I and *Hin*d III at 37 °C overnight, and subcloned into pET28a vector (pET28-Har-Relish). The recombinant Har-Relish protein was expressed in BL21 cells induced by 1 mM IPTG for 9 h. The *E. coli* pellet was solubilized in 6 M urea in 50 mM Tris-HCl buffer, pH 8.0, followed by Ni-NTA column purification. Purified recombinant Har-Relish protein was used to generate polyclonal antibodies in rabbit with subcutaneous injection.

**Northern blot, Competitive RT-PCR and Southern blot analysis**

Total RNA from fat body was obtained using an acid guanidinium thiocyanate-phenol-chloroform method [2]. Total RNA (35 µg) was separated on a 1.2% formaldehyde-agarose gel and the RNA was visualized by staining with ethidium bromide and photographed under UV light. RNA was then transferred to Hybond N^+^ membrane (Amersham). The membrane was hybridized using a ^32^P-labelled probe, which was made with Har-Relish cDNA and a Random Primer DNA Labeling Kit (TaKaRa). After prehybridization for 6 h in 5× sodium chloride-sodium phosphate-EDTA (SSPE) [1× SSPE=180 mM sodium chloride, 10 mM sodium phosphate, pH 7.7, 1 mM EDTA] containing 50% formamide, 5× Denhardt’s solution, 0.1% SDS and 100 μg/ml denatured salmon sperm DNA, the probe was added. After 24 h hybridization at 42 °C, the nylon membrane was washed with 0.2×SSPE at 65 °C and exposed to X-ray film for 24 h at -80 °C.

Developmental expression of Har-CL mRNA in the fat body was determined by combination of competitive RT-PCR and Southern blot analysis [3]. Briefly, truncated Har-CL (T-Har-CL) and Har-Relish (T-Har-Relish) cDNA plasmids were constructed and transcribed *in vitro* to mRNA as a standard for semi-quantitative PCR. T-Har-CL (2 ng) or T-Har-Relish (1 ng) mRNA and total RNA (2 μg) were mixed and reverse-transcribed with specific primers, RTL (5’-TTCGGTATAGGAGCGGAT-3’) for Har-CL and RXR (5’-CGGAATTCCTTCTTATGACACGTGCCGC-3’) for Har-Relish, in a total volume of 25 μl. One microlitre of the first strand cDNA was used for PCR amplification. Two specific primers, HLF (5’-GAGTGGAGCGCCTTCAAG-3’) and HLR (5’-CCGTGGTCGAGGTCTGTGGAG-3’), were used to amplify the control T-Har-CL and target Har-CL cDNAs in the same PCR. Similarly, two specific primers, N1F (5’-CGATCCCGATCCGACTTCG-3’) and N1R (5’-CCGGCTTCTAACTTCAGCC-3’), were used for the control T-Har-Relish and target Har-Relish cDNAs in the same PCR. PCR amplification was performed in a 25 μl reaction mixture under the following conditions: 5 min at 94 °C; 24 cycles for Har-CL or 26 cycles for Har-Relish of 1 min at 94 °C, 1 min at 60 °C and 1 min at 72 °C; and 10 min at 72 °C. PCR products were separated on 1.5% agarose and transferred on to Hybond-N^+^ nylon membrane. Hybridization, washing and signal detection of the blots were similar to Northern blots.

**Genome walking**

The genomic DNA of *H. armigera* was extracted from the 6^th^ instar larvae according to the previous method [4], and the DNA sample was then treated with Genome Walker^TM^ Universal Kit (Clontech) according to the manufacturer’s protocol. The primers for primary PCR CLW1 (5’-ACCACGCACAGCAGCACCGC-3’) and secondary PCR CLW2 (5’-CCGCGTCGCAATCGTTTCTGC-3’) were designed based on Har-CL cDNA sequence [3]. The samples were denatured at 94 °C for 10 min, followed by a 30 cycles reaction 1) primary PCR: 94 °C for 30 s; 57 °C for 30 s; 72 °C for 4 min; 2) secondary PCR: 94 °C for 30 s; 60 °C for 30 s; 72 °C for 4 min. The PCR products were separated by 1.0% agarose gel electrophoresis, and then purified, ligated into the pMD18-T vector (TaKaRa) for sequencing.

**Cloning of Relish and Dorsal cDNAs**

One microgram of total RNA was reverse transcribed at 42 °C for 1 h in a volume of 25 μl with the M-MLV reverse transcription system (Promega). One microliter of the reverse transcription product was added to 50 μl of the PCR reaction system. Amplification was performed with degenerate primers DP1 (5’-ATGGGTAT(C/A)AT(A/T)CACAC(A/T)GC-3’) and DP2 (5’-TTTTTGTT(A/G)AC(T/C)TT(T/C)TC(G/C)AC-3’) for *Relish*, and degenerate primers DP3 (5’-CCCCACAACCT(A/T/G/C)G(A/T/G/C)GG-3’) and DP4 (5’-CGCTCATGGCCTT(C/T)TT(G/A)TC-3’) for *Dorsal*, these degenerate primers were designed according to the conserved *Relish* or *Dorsal* cDNA sequences from *D. melanogaster* and *B. mori* [5-8]. PCR was performed under the following conditions: 30 s at 94 °C, 30 s at 48 °C, 30 s at 72 °C with 30 cycles, and then 10 min at 72 °C. A 356 bp product for Relish and a 332 bp product for Dorsal were obtained and sequenced.

For 5′- and 3′-RACE, the first strand cDNA (Fs-cDNA) was synthesized using the SMART RACE cDNA amplification kit, according to the manufacturer’s protocols (Clontech). Two primers RR1R (5’-GAAGACATCTTCTCCCCCAG-3’) and RR2R (5’-GCACTGCCGGAGGACCGAC-3’) for Relish 5’-RACE, and two primers RR1F (5’-AGGATGTGGCTGCGCTAC-3’) and RR2F (5’-GTACAATGCGAGAATATGGCC-3’) for *Relish* 3’-RACE, and two primers DR1R (5’-CGTCGGAGACGACCGGCGCC-3’) and DR2R (5’-CCGCCCCGAGTCGTCCGGC-3’) for Dorsal 5’-RACE and two primers DR1F (5’-TCATGAACGCTGCGAGCGAGG-3’) and DR2F (5’-GAGTCTGCACGATCCCGACCC-3’) for *Dorsal* 3’-RACE were respectively synthesized. Using nest PCR, 5’- and 3’-cDNAs of Relish and Dorsal were respectively amplified.

**Construction of reporter gene and deletion mutagenesis**

A series of stepwise deletion fragments of Har-CL promoter starting at position +30 and extending to -256, -345, -522, -643, -1292, -1554, -1828 and -1911 bp were generated by PCR, the forward primers and a common reverse primer as indicated in Table S1. The eight DNA fragments were ligated into the pMD18-T vectors (TaKaRa), and sequenced. The fragments were then cut out from pMD18-T plasmids with *Nhe* I and *Kpn* I, and ligated to luciferase reporter plasmid pGL3-basic vectors (Promega).

We used MutanBEST Kit (TaKaRa) to obtain a series deletion mutant reporter gene plasmid as described in the manufacturer’s manual, and the primers were indicated in Table S1.

**Construction of the overexpression system**

Har-Relish ORF and Har-Rel-D were respectively amplified with primers RXF (5’-CGGGATCCATGTCTACAAGCAGTGACCACG-3’) and RXR (5’-CGGAATTCGCGGGTCTTGTCAATCG) for Relish, and primers RXF and RdXR (5’-CGGAATCTTGCTGTAACTTTGGCC-3’) for Rel-D. Har-Dorsal ORF was amplified with primers DXF (5’-CGGGATCCATGGCGCGCCGCGACCAG-3’) and DXR (5’-CGGAATTCGTTCAGTAGTCGAGTGCGGGC-3’). These primers contained *Bam*H I and *Eco*R I restriction sites, respectively. The PCR products were purified, digested with *Bam*H I and *Eco*R I, and subcloned directly into the digested blank plasmids (pIZ/V5-His). Then, we added a GFP label into the vector to detect the transfection efficiency.

**Chromatin immunoprecipitation (ChIP) assay**

The ChIP assay was performed as described previously in Bao et al. [9]. Fat bodies of wandering larvae was placed in 1 ml of nuclei extraction buffer (0.5% Triton X-100, 10 mM Tris-HCl, pH 7.5, 3 mM CaCl_2_, 0.25 M sucrose, 1×protease inhibitor cocktail, 1 mM DTT, and 0.2 mM PMSF). After homogenate on ice, formaldehyde was added to a 1% final concentration. After 15 min rotation, 1.25 M glycine was added and rotation at room temperature for 5 min to stop the reactivity. Then the nuclei was collected and resuspended in 300 µl of SDS lysis buffer (1% SDS, 10 mM EDTA, 50 mM Tris-HCl, pH 8.1). The chromatin was sheared to approximately 200 to 1000 bp fragments by sonication and then centrifuged at 12,000 g for 15 min at 4 °C. Anti-Har-Relish was used for immunoprecipitation, pre-immune serum as empty control, and another antibody (anti-Har-CL) as mock. After rotation of the immunoprecipitation samples at 4 °C over 12 h, the beads were washed. Then, 100 µl elution buffer was added and put it at 65 °C for 4 h. This step also included the input samples. The DNA was purified by phenol/chloroform extraction, and PCR was performed for 30 cycles with primers CLPCF (5’-AAATCCTCCAACTCCCTCCAAGC-3’) and CLPCR (5’-GTCCGACATAAGCATAAG CATCG -3’), using the following conditions: 45 s at 94 °C, 45 s at 58 °C, 50 s at 72 °C.

**DsRNA generation**

Double-stranded (ds) RNAs were synthesized from PCR templates using the T7 RiboMAX™ Express RNAi System (Promega). PCRs were performed with primers which contained a T7 promoter at the 5′ end. To generate the dsRNA of Har-Relish, the primers were RITF (5’-TAATACGACTCACTATAGGGGAGTGCATCGACGAAGACG-3’) and RIR (5’-CTCAACATCGAACATGCAAC-3’) for sense RNA synthesizing, RITR (5’-TAATACGACTCACTATAGGGCTCAACATCGAACATGCAAC-3’) and RIF (5’-GAGTGCATCGACGAAGACG-3’) for anti-sense RNA synthesizing. To generate the dsRNA of Har-EcR, the primers were T7EcRF (5’-TAATACGACTCACTATAGGCGCTGGTATAACAACGGAGG-3’) and EcRR (5’-AGCTGGAGACAACTCCTCACG-3’) for sense RNA synthesizing, EcRF (5’-CGCTGGTATAACAACGGAGG-3’) and T7EcRR (5’-TAATACGACTCACTATAGGAGCTGGAGACAACTCCTCACG-3’) for anti-sense RNA synthesizing. To generate the dsRNA of Har-CL, the primers were T7CLF (5’-TAATACGACTCACTATAGGGCCTTCAAGTACATCAAG-3’) and CLR (5’-TCTTGATGTAGCCGAGGT-3’) for sense RNA synthesizing, CLF (5’-GCCTTCAAGTACATCAAG-3’) and T7CLR (5’-TAATACGACTCACTATAGGTCTTGATGTAGCCGAGGT-3’) for anti-sense RNA synthesizing. HzAM1 cells were seeded into 24-wells plate at 10^5^ cells per well. 4 μg/mL dsRNAs of the Har-Relish were transfected into cells as above. When cultured for 48 h, 20E and 10% FBS were then added. After 24 h, total RNA was extracted and RT-PCRs were performed using the following condition: 30 s at 94 °C, 30 s at 65 °C, 30 s at 72°C.

**References**

1. Uchida K, Ohmori D, Ueno T, Nishizuka M, Eshita Y, et al. (2001) Preoviposition activation of cathepsin-like proteinases in degenerating ovarian follicles of the mosquito *Culex pipiens pallens*. Dev Biol 237: 68-78.

2. Chomczynski P, Sacchi N (1987) Single-step method of RNA isolation by acid guanidinium thiocyanate-phenol-chloroform extraction. Anal Biochem 162: 156-159.

3. Liu J, Shi GP, Zhang WQ, Zhang GR, Xu WH (2006) Cathepsin L function in insect moulting: molecular cloning and functional analysis in cotton bollworm, *Helicoverpa armigera*. Insect Mol Biol 15: 823-834.

4. Ohshima Y, Suzuki Y (1977) Cloning of the silk fibroin gene and its flanking sequences. Proc Natl Acad Sci USA 74: 5363-5367.

5. Steward R (1987) Dorsal, an embryonic polarity gene in *Drosophila*, is homologous to the vertebrate proto-oncogene, c-rel. Science 238: 692-694.

6. Stoven S, Ando I, Kadalayil L, Engstrom Y, Hultmark D (2000) Activation of the *Drosophila* NF-kappaB factor Relish by rapid endoproteolytic cleavage. EMBO Rep 1: 347-352.

7. Tanaka H, Matsuki H, Furukawa S, Sagisaka A, Kotani E, et al. (2007) Identification and functional analysis of Relish homologs in the silkworm, *Bombyx mori*. Biochim Biophys Acta 1769: 559-568.

8. Tanaka H, Yamamoto M, Moriyama Y, Yamao M, Furukawa S, et al. (2005) A novel Rel protein and shortened isoform that differentially regulate antibacterial peptide genes in the silkworm *Bombyx mori*. Biochim Biophys Acta 1730: 10-21.

9. Bao B, Xu WH (2011) Identification of gene expression changes associated with the initiation of diapause in the brain of the cotton bollworm, *Helicoverpa armigera*. BMC Genomics 12: 224.
